# Development and Validation of a Machine Learning Model to Estimate Bacterial Sepsis Among Immunocompromised Recipients of Stem Cell Transplant

**DOI:** 10.1001/jamanetworkopen.2021.4514

**Published:** 2021-04-19

**Authors:** Margaret L. Lind, Stephen J. Mooney, Marco Carone, Benjamin M. Althouse, Catherine Liu, Laura E. Evans, Kevin Patel, Phuong T. Vo, Steven A. Pergam, Amanda I. Phipps

**Affiliations:** 1 Department of Epidemiology, University of Washington, Seattle; 2 Vaccine and Infectious Disease Division, Fred Hutchinson Cancer Research Center, Seattle, Washington; 3 Harborview Injury Prevention and Research Center, Seattle, Washington; 4 Department of Biostatistics, University of Washington, Seattle; 5 Department of Statistics, University of Washington, Seattle; 6 Institute for Disease Modeling, Bellevue, Washington; 7 Information School, University of Washington, Seattle; 8 Department of Biology, New Mexico State University, Las Cruces; 9 Division of Allergy and Infectious Disease, Department of Medicine, University of Washington, Seattle; 10 Clinical Research Division, Fred Hutchinson Cancer Research Center, Seattle, Washington; 11 Antimicrobial and Outpatient Parenteral Antimicrobial Therapy Program, Seattle Cancer Care Alliance, Seattle, Washington; 12 Division of Pulmonary, Critical Care and Sleep Medicine, University of Washington, Seattle; 13 Oncology and Bone Marrow Transplant Intensive Care Unit, University of Washington, Seattle; 14 Medical Intensive Care Unit, University of Washington, Seattle; 15 Division of Medical Oncology, University of Washington, Seattle; 16 School of Medicine, University of Washington, Seattle; 17 Public Health Sciences Division, Fred Hutchinson Cancer Research Center, Seattle, Washington

## Abstract

**Importance:**

Sepsis disproportionately affects recipients of allogeneic hematopoietic cell transplant (allo-HCT), and timely detection is crucial. However, the atypical presentation of sepsis within this population makes detection challenging, and existing clinical sepsis tools have limited prognostic value among this high-risk population.

**Objective:**

To develop a full risk factor (demographic, transplant, clinical, and laboratory factors) and clinical factor–specific automated bacterial sepsis decision support tool for recipients of allo-HCT with potential bloodstream infections (PBIs).

**Design, Setting, and Participants:**

This prognostic study used data from adult recipients of allo-HCT transplanted at the Fred Hutchinson Cancer Research Center, Seattle, Washington, between June 2010 and June 2019 randomly divided into 70% modeling and 30% validation data sets. Tools were developed using the area under the curve (AUC) optimized SuperLearner, and their performance was compared with existing clinical sepsis tools: National Early Warning Score (NEWS), quick Sequential Organ Failure Assessment (qSOFA), and Systemic Inflammatory Response Syndrome (SIRS), using the validation data set. Data were analyzed between January and October of 2020.

**Main Outcomes and Measures:**

The primary outcome was high–sepsis risk bacteremia (culture confirmed gram-negative species, *Staphylococcus aureus*, or *Streptococcus spp* bacteremia), and the secondary outcomes were 10- and 28-day mortality. Tool discrimination and calibration were examined using accuracy metrics and expected vs observed probabilities.

**Results:**

Between June 2010 and June 2019, 1943 recipients of allo-HCT received their first transplant, and 1594 recipients (median [interquartile range] age at transplant, 54 [43-63] years; 911 [57.2%] men; 1242 individuals [77.9%] identifying as White) experienced at least 1 PBI. Of 8131 observed PBIs, 238 (2.9%) were high–sepsis risk bacteremia. Compared with high–sepsis risk bacteremia, the full decision support tool had the highest AUC (0.85; 95% CI, 0.81-0.89), followed by the clinical factor–specific tool (0.72; 95% CI, 0.66-0.78). SIRS had the highest AUC of existing tools (0.64; 95% CI, 0.57-0.71). The full decision support tool had the highest AUCs for PBIs identified in inpatient (0.82; 95% CI, 0.76-0.89) and outpatient (0.82; 95% CI, 0.75-0.89) settings and for 10-day (0.85; 95% CI, 0.79-0.91) and 28-day (0.80; 95% CI, 0.75-0.84) mortality.

**Conclusions and Relevance:**

These findings suggest that compared with existing tools and the clinical factor–specific tool, the full decision support tool had superior prognostic accuracy for the primary (high–sepsis risk bacteremia) and secondary (short-term mortality) outcomes in inpatient and outpatient settings. If used at the time of culture collection, the full decision support tool may inform more timely sepsis detection among recipients of allo-HCT.

## Key Points

**Question:**

Can machine learning be used with electronic medical record data to improve bacterial sepsis prediction among recipients of allogeneic hematopoietic cell transplant (allo-HCT)?

**Findings:**

In this prognostic study including 1943 recipients of allo-HCT, the population-specific full predictor bacterial sepsis decision support tool (SHBSL) had superior prognostic performance regardless of outcome or patient location compared with the clinical factor–specific SHBSL and existing tools. Additionally, SHBSL had higher positive predictive values relative to sensitivities than existing tools.

**Meaning:**

These findings suggest that, if used at the time of blood culture collection, the SHBSL could provide relevant information regarding bacterial sepsis risk and antibiotic needs of recipients of allo-HCT.

## Teaser

This prognostic study uses machine learning and electronic medical record data to develop and test an automated bacterial sepsis decision support tool for immunocompromised recipients of allogeneic hematopoietic cell transplant.

## Introduction

Sepsis disproportionately affects immunocompromised populations, including recipients of allogeneic hematopoietic cell transplant (allo-HCT).^1^ Early empirical broad-spectrum antibiotic use among recipients of HCT with bacterial sepsis has been associated with 3-fold reduced risk of 30-day mortality.^2^ However, antibiotic use is associated with increased antibiotic resistance, hospital-acquired infections, and, in recipients of HCT, may have detrimental microbiota-mediated associations with mortality.^3,4,5,6,7,8^ To minimize these outcomes, early and accurate sepsis detection among recipients of allo-HCT is crucial.

However, sepsis is challenging to diagnose among recipients of allo-HCT. In individuals who are immunosuppressed, sepsis has less common presentations, manifests with subtle clinical findings, and progresses more rapidly than in immunocompetent populations.^9^ Further, common complications of HCT, such as engraftment syndrome, anemia, and drug adverse effects, can present with similar signs and symptoms as sepsis.^9,10^ Unsurprisingly, current clinical sepsis tools, which were built among patients from the general population, have limited prognostic value among recipients of allo-HCT, and their use may lead to missed sepsis events and antibiotic misuse.^11^

With the goal of improving the accuracy of bacterial sepsis risk estimation at the time of clinical infection consideration (blood culture collection) in inpatient and outpatient settings, we developed 2 automated bacterial sepsis decision support tools for recipients of allo-HCT with potential bloodstream infections (PBIs) using a state-of-the-art stacked ensembling technique, the SuperLearner.^12^ Our tools, super HCT bacterial sepsis learner (SHBSL) and clinical factor–specific super HCT bacterial sepsis learner (C-SHBSL), have the potential to be integrated into electronic medical record (EMR) systems and to provide treatment decision support in the form of patient-specific sepsis risk probability estimates or flags for patient who are at high risk at the time of blood culture collection (eFigure 1 in the Supplement). Through retrospective evaluation, we assessed whether our population specific tools led to more accurate risk estimation among recipients of allo-HCT than commonly used existing tools.

## Methods

This prognostic study was approved by the Fred Hutchinson Cancer Research Center (FHCRC) institutional review board. All participants provided written informed consent. This study followed the Transparent Reporting of a Multivariable Prediction Model for Individual Prognosis or Diagnosis (TRIPOD) reporting guidelines.

### Study Overview, Population, and Data Collection

We identified adult (ie, age ≥18 years) recipients of allo-HCT who agreed to research participation and received their first transplant at the FHCRC or Seattle Cancer Care Alliance between June 2010 and June 2019. Data from the initial 100 days after transplant, a period of close patient monitoring by inpatient and outpatient transplant teams, or until patient death, if prior to day 100, were retrospectively collected.

Data were queried from a prospectively maintained transplant database and EMRs. We defined outliers as measurements outside of biologically plausible ranges and, following a EMR review verification of a randomly selected 10% of outliers, dropped outliers prior to missing value imputation.

### Cohort Development

We developed and evaluated the tools among all PBIs that occurred within the study. We defined PBI as any blood culture, including surveillance cultures, collected in the absence of recent (ie, past 3 days) antibiotic administration or prescription other than the center recommended neutropenia prophylaxis (ie, levofloxacin). We considered multiple cultures collected within 1 hour to be the same event (culture time defined as time of first nonsurveillance culture) and excluded follow-up cultures, defined as cultures collected continuously (within 36 hours of prior culture) for the 14 days after a culture with positive results or until distinct organisms were identified, whichever came first. Patients for whom any PBIs were identified were randomly divided into a modeling (70%) or validation (30%) group, and PBIs were assigned to the modeling or validation data set based on the patient’s grouping.

### Prophylaxis and Culture Collection Guidelines

FHCRC guidelines during the study have been described elsewhere.^11^ Briefly, cultures were recommended for patients with fevers, and surveillance cultures were recommended weekly or biweekly for patients receiving high-dose glucocorticoids (ie, >0.5 mg/kg). Levofloxacin was used as the primary antibiotic prophylaxis agent during neutropenic periods while patients with neutropenic fever received empirical therapy with ceftazidime, cefepime, or meropenem.

### Outcome and Factors

The atypical presentation of sepsis among recipients of HCT prevented us from using a symptom-based primary end point.^13,14^ Instead, we selected bacteremia with high risk for sepsis (ie, blood culture–confirmed gram-negative organisms, *Staphylococcus aureus*, or *Streptococcus spp* bacteremia) based on evidence that sepsis is significantly more likely among recipients of HCT with PBIs and the selected bloodstream infections than without (eAppendix 1 in the Supplement).^15^

Because our end point excludes sepsis events with negative results in blood cultures,^16,17^ we feared our tools may capture bacteremia but not sepsis risk. To test the ability of our tools to capture sepsis risk in general, not just bacteremia risk, we examined 2 short-term mortality secondary outcomes (ie, 10-day and 28-day mortality) under the assumption that mortality was more common among patients with sepsis than without.^18^ However, unlike a 2016 study by Seymour et al,^18^ we chose against a primary mortality end point because we were unable to account for the association between mortality and antimicrobial and supportive treatment undergone by most patients in our retrospectively collected sample.^19,20,21^

We considered bedside assessable demographic and clinical factors as potential factors in C-SHBSL. For SHBSL, we additionally included a wide array of comorbidities, transplant factors, biomarkers, vitals, and recent infection data (eTable 1 in the Supplement). Additionally, in SHBSL, we included binary indicators of measurement missingness and, to account for trends in numerical factors during the window of measurement collection, linear change-over-time factors developed using univariable linear regressions.

Factors were standardized to a uniform unit (eg, weight in kg), and lesser- or greater-than values were converted to the first number included in the statement (eg, 35 °C for ≤35 °C). Temperatures were standardized to oral temperatures by adding 0.45 °C to axillary measurements and subtracting 0.2 °C from arterial measurements.^22^ Binary (0 or 1) variables were developed from categorical factors, all of which were nominal. Each factor was standardized to a mean of zero and SD of 1.

Data for the 24 hours prior to and 2 hours after culture collection were extracted, and the measurement taken closest in time to collection was retained. Potential factors with more than 35% missingness were excluded. Missingness and change-over-time factors were included if the static measurement was included. Missing observations for frequently captured factors were filled using a single normal value imputation approach (eAppendix 2, eFigure 2, and eTable 2 in the Supplement).^18,23,24,25^

### Tool Development

We developed our tools in the modeling data set using the Super Learner with 10-fold cross-validation, optimized with respect to area under the receiver operating characteristic curve (AUROC). The Super Learner is a statistical learning technique that uses cross-validation to construct an optimal ensemble (weighted mean) of user-supplied candidate algorithms.^26^ The resulting model, the Super Learner, has been proven in large samples to perform as well as the best performing ensemble of specified algorithms and shown in small samples to perform essentially as well as the best performing ensemble of specified algorithms.^26,27,28^ To optimize the resulting Super Learners, our library included a broad selection of parametric, semiparametric, and nonparametric learners and both linear and nonlinear algorithms. Additionally, our library included hyperparameter and screening algorithm variants (eAppendix 3 in the Supplement). As with the modeling and validation randomization, observations were partitioned into cross-validation folds at the patient level.

### Statistical Analysis

#### Tool Calibration and Discrimination

Using the validation data set, we compared the calibration and discrimination of our tools to 3 existing clinical sepsis tools available for use in outpatient settings: the National Early Warning Score (NEWS), quick Sequential Organ Failure Assessment (qSOFA), and Systemic Inflammatory Response Syndrome (SIRS). We assessed calibration by comparing the mean estimated vs observed outcome probabilities and examined calibration plots.^29,30,31,32^ To evaluate discrimination, we compared AUROCs, area under the precision and recall curve (pr-AUC), categorical net reclassification index (NRI) (eAppendix 4 in the Supplement), and predictive accuracy metrics (ie, sensitivity, specificity, negative predictive value [NPV], and positive predictive value [PPV]) for all cut points (primary analysis), and we compared qSOFA/SIRS at a cut point of 2 or greater, NEWS at cut points of 4 or greater and 7 or greater, and SHBSL/C-SHBSL (primary analysis selected optimal [Upper-Left Method^33^] cut points) for secondary and sensitivity analyses. Optimal cut points (upper-left) were identified for the primary and secondary analyses. To visually compare tool discrimination, we generated ROC and precision and recall curves.

We performed secondary analyses assessing tool performance by inpatient status at time of culture collection and regarding short-term (10- and 28-day) mortality.^18,34^ Inpatient status was defined for each calendar day and patients were considered inpatient for the full day of hospital admission, discharge and during intensive care unit (ICU) stays. Additionally, we estimated the cross-validated AUROCs of our tools using 10-fold nested cross-validation and the bootstrapped AUROCs of existing tools using 1000 modeling and validation split variants. All analyses were performed using R statistical software version 3.6.2 (R Project for Statistical Computing) base functions and functions from the *rms*, *fbroc*, *OptimalCutpoints*, and *SuperLearner* packages.^12,32,35,36^ *P* values were 2-sided, and statistical significance was set at α = .05. Data were analyzed from between January and October of 2020.

#### Sensitivity Analyses

We performed several sensitivity analyses to examine the generalizability of our tools. Specifically, we explored whether our evaluation results were robust to varying demographic characteristic, missingness, recent antibiotic use, data collection, and culture collection and inclusion scenarios. The sensitivity analyses were performed using the validation data set and are described in eAppendix 6 in the Supplement.

## Results

### Study Population and Cohort

Between June 2010 and June 2019, 1943 individuals received their first allo-HCT at the Seattle Cancer Care Alliance or FHCRC, and 1594 individuals (median [interquartile range] age at transplant, 54 [43-63] years; 911 [57.2%] men; 1242 individuals [77.9%] identifying as White) experienced at least 1 PBI (Table 1; eFigure 3 in the Supplement). During follow-up, these patients experienced 8131 PBIs, including 6046 PBIs (74.4%) identified in outpatient settings and 2085 PBIs (25.6%) in inpatient settings (eFigure 4 in the Supplement). Patients who developed high-risk bacteremia, compared with those who did not, were more likely to experience grade 4 acute graft-vs-host disease (19 patients [9.8%] vs 38 patients [2.7%]) and die during follow-up (33 patients [17.1%] vs 105 patients [7.5%]) (Table 1).

**Table 1.** Baseline Characteristics of Participants Stratified by Occurrence of High-Risk Bacteremia

Characteristic	No. (%)		
	Full population (N = 1594)	High-risk bacteremia	
		Yes (n = 193)	No (n = 1401)
Calendar year at transplant, median (IQR)	2014 (2012-2016)	2013 (2011-2016)	2014 (2012-2016)
Age at transplant, median (IQR)	54 (43-63)	53 (41-62)	55 (43-63)
Sex			
Men	911 (57.2)	107 (55.4)	804 (57.4)
Women	683 (42.8)	86 (44.6)	597 (42.6)
Race/ethnicity			
American Indian or Alaskan Native^a^	24 (1.5)	2 (1.0)	22 (1.6)
Asian^a^	119 (7.5)	16 (8.3)	103 (7.4)
Black^a^	25 (1.6)	5 (2.6)	20 (1.4)
Multiple^a^	22 (1.4)	5 (2.6)	17 (1.2)
Native Hawaiian or other Pacific Islander^a^	25 (1.6)	5 (2.6)	20 (1.4)
Unknown^a^	54 (3.4)	5 (2.6)	49 (3.5)
White^a^	1242 (77.9)	141 (73.1)	1101 (78.6)
Hispanic or Latino	83 (5.2)	14 (7.3)	69 (4.9)
Underlying disease			
Acute leukemia			
Lymphoblastic	212 (13.3)	34 (17.6)	178 (12.7)
Myelogenous	580 (36.4)	64 (33.2)	516 (36.8)
Myelodysplastic syndromes	410 (25.7)	48 (24.9)	362 (25.8)
Other	392 (24.6)	47 (24.4)	345 (24.6)
Cell donor type			
Bone marrow	126 (7.9)	20 (10.4)	106 (7.6)
Bone marrow, peripheral blood stem cells	3 (0.2)	1 (0.5)	2 (0.1)
Umbilical cord blood	179 (11.2)	31 (16.1)	148 (10.6)
Peripheral blood stem cells	1286 (80.7)	141 (73.1)	1145 (81.7)
Donor relation			
Child	38 (2.4)	6 (3.1)	32 (2.3)
Half sibling	5 (0.3)	0 (0)	5 (0.4)
Not related	1107 (69.4)	135 (69.9)	972 (69.4)
Parent	17 (1.1)	4 (2.1)	13 (0.9)
Relative	1 (0.1)	0 (0)	1 (0.1)
Sibling	426 (26.7)	48 (24.9)	378 (27.0)
Acute graft-vs-host disease grade^b^			
0	328 (20.6)	32 (16.6)	296 (21.1)
1	78 (4.9)	7 (3.6)	71 (5.1)
2	952 (59.7)	97 (50.3)	855 (61.0)
3	179 (11.2)	38 (19.7)	141 (10.1)
4	57 (3.6)	19 (9.8)	38 (2.7)
High-risk bacteremia^b^^,^^c^			
Gram-negative species	104 (6.5)	104 (53.9)	NA
* Staphylococcus aureus*	8 (0.5)	8 (4.1)	NA
* Streptococcus spp*	87 (5.5)	87 (45.1)	NA
Neutropenia prophylaxis^d^	1439 (90.3)	178 (92.2)	1261 (90.0)
Died^b^	138 (8.7)	33 (17.1)	105 (7.5)

Abbreviations: IQR, interquartile range; NA, not applicable.

^a^ Non-Hispanic.

^b^ During follow-up.

^c^ A total of 6 patients experienced more than 1 type of high-risk bacteremia event and appear in 2 rows.

^d^ Defined as administration or prescription of levofloxacin during periods of neutropenia (absolute neutrophil count, <500/μL [to convert to ×10^9^/L, multiply by 0.001]) during follow-up.

Of 8131 PBIs, 238 (2.9%) were defined as high–sepsis risk bacteremia, 258 (3.2%) occurred with 28 days of death, and 74 (0.9%) occurred within 10 days of death. The modeling data set contained 5845 PBIs, of which 168 (2.9%) high–sepsis risk bacteremia. The validation data set included 2286 PBIs, of which 70 PBIs (3.1%) were high–sepsis risk bacteremia. Patients randomized to the modeling and validation groups had similar demographic and medical characteristics (eTable 3 in the Supplement). Of 35 vital measurements or biomarkers examined, 17 were discarded owing to excessive missingness (ie, >35%; eTable 1 in the Supplement).

### Tool Calibration and Discrimination

**Figure 1.**
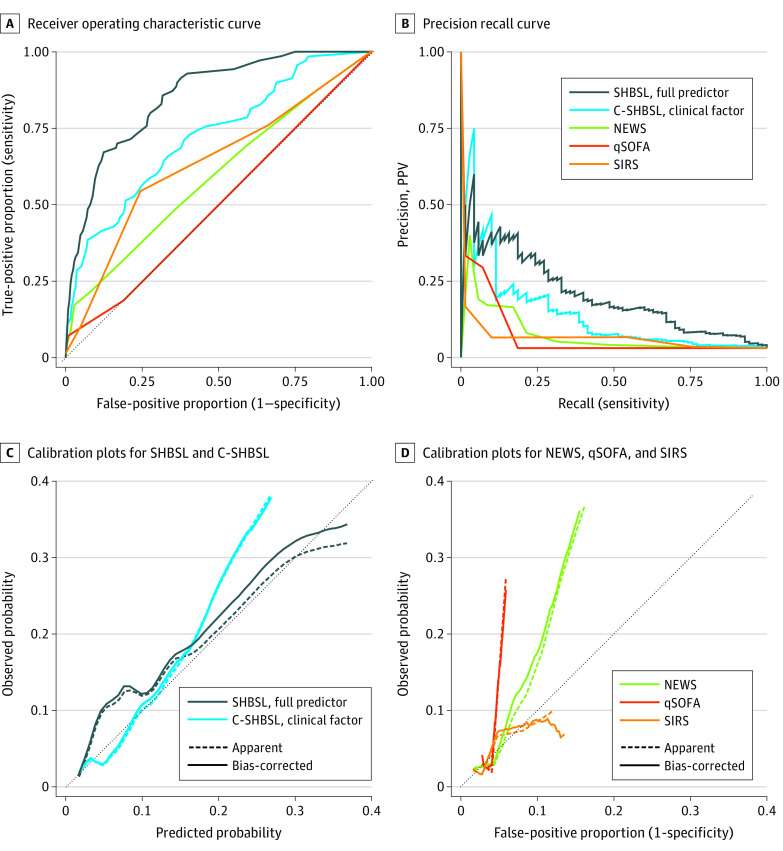
Receiver Operating Characteristic Curves, Precision Recall Curve, and Calibration Plots for High–Sepsis Risk Bacteremia for All Potential Bloodstream Infections Among Recipients of Allogeneic Hematopoietic Cell Transplant C- indicates clinical factor–specific; NEWS, National Early Warning Score; PPV, positive predictive value; qSOFA, quick Sequential Organ Failure Assessment; SHBSL, super hematopoietic cell transplant bacterial sepsis learner; and SIRS, Systemic Inflammatory Response Syndrome. High–sepsis risk bacteremia was defined as gram-negative, *Staphylococcus aureus*, or *Streptococcus* bacteremia.

In detecting patients with high-risk bacteremia, SHBSL had the highest AUROC (0.85; 95% CI, 0.81-0.89) and pr-AUC (0.20), and C-SHBSL had the second highest (AUROC: 0.72; 95% CI, 0.66-0.78; pr-AUC: 0.13). qSOFA had the lowest AUROC (0.51, 95% CI, 0.46-0.56) and all 3 existing tools had pr-AUCs of 0.07 (Table 2, Figure 1A and B). At optimal cut points, SHBSL had a sensitivity of 80.0% (95% CI, 69.2%-87.7%) and a specificity of 72.8% (95% CI, 69.5%-75.8%), and C-SHBSL had a sensitivity of 65.7% (95% CI, 51.9%-77.3%) and a specificity of 66.9% (95% CI, 64.0%-69.7%) (Table 2). At cut points with sensitivities of 90% or greater, SHBSL had a PPV of 7.3% or less and C-SHBSL had a PPV of 4% or less (eTable 4 in the Supplement). All tools had high NPVs (range, 96.9%-100%) (eTable 4 in the Supplement). Compared with the other tools, SHBSL had significantly better risk classification (C-SHBSL: NRI, 0.37; 95% CI, 0.20 - 0.54; *P* < .001; SIRS: NRI, 0.52; 95% CI, 0.34-0.69; *P* < .001; qSOFA: NRI, 0.31; 95% CI, 0.13-0.49; *P* < .001; NEWS: NRI, 0.35; 95% CI, 0.20-0.49; *P* < .001) (eTable 5 in the Supplement).

**Table 2.** Discrimination and Predictive Accuracy of Sepsis Tools for High–Sepsis Risk Bacteremia Among Recipients of Allogeneic Hematopoietic Cell Transplant With Potential Bloodstream Infections

Measure	AUROC^a^	pr-AUC	% (95%CI)				Upper-left cut point^c^
			Sensitivity^b^	Specificity^b^	Predictive value^b^		
					Positive	Negative	
Clinical factor–specific SHBSL^d^	0.72 (0.66-0.78)	0.13	65.7 (51.9-77.3)	66.9 (64.0-69.7)	5.9 (4.4-7.9)	98.4 (97.3-99.1)	9.3%
Full SHBSL^d^	0.85 (0.81-0.89)	0.20	80.0 (69.2-87.7)	72.8 (69.5-75.8)	8.5 (6.5-11)	99.1 (98.4-99.5)	5.0%
SIRS (≥2)	0.64 (0.57-0.71)	0.07	54.3 (41.9-66.2)	75.9 (73.6-78.0)	6.6 (4.8-9.1)	98.1 (97.1-98.8)	2
qSOFA (≥2)	0.51 (0.46-0.56)	0.07	7.1 (3.0-16.1)	99.5 (99.0-99.7)	29.4 (13.2-53.3)	97.1 (96.2-97.9)	1
NEWS	0.6 (0.52-0.67)	0.07	NA	NA	NA	NA	3
Cut point of ≥4	NA	NA	31.4 (21.5-43.4)	81.9 (80.1-83.6)	5.2 (3.4-7.8)	97.4 (96.4-98.2)	NA
Cut point of ≥7	NA	NA	8.6 (3.9-17.9)	98.7 (98.0-99.1)	17.1 (7.8-33.7)	97.2 (96.2-97.9)	NA

Abbreviations: AUROC, area under the receiver operating characteristic curve; NA, not applicable; NEWS, National Early Warning Score; pr-AUC, area under the precision recall curve; qSOFA, quick Sequential Organ Failure Assessment; SHBSL; super hematopoietic cell transplant bacterial sepsis learner; SIRS, Systemic Inflammatory Response Syndrome.

^a^ 95% CI estimated using 2000 stratified bootstrapped replicates.

^b^ 95% CI estimated using generalized estimating equations with robust SEs.

^c^ Risk-probability cut point closest to the 01 (upper left) corner of the receiver operating characteristic curve.

^d^ Based on upper-left selected cut points from primary validation analysis (clinical factor–specific SHBSL: 9.3%; SHBSL: 5.0%).

**Figure 2.**
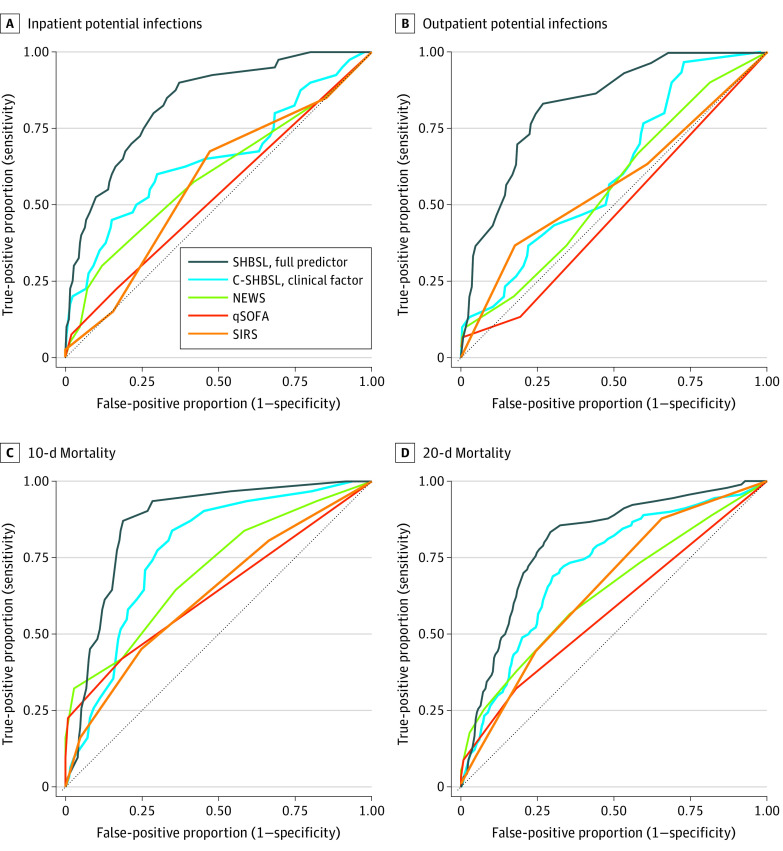
Receiver Operating Characteristic Curves for High-Risk Bacteremia Among Inpatient and Outpatient Identified Potential Bloodstream Infections and for 10-Day and 28-Day Mortality Among All Potential Bloodstream Infections C- indicates clinical factor–specific; NEWS, National Early Warning Score; qSOFA, quick Sequential Organ Failure Assessment; SHBSL, super hematopoietic cell transplant bacterial sepsis learner; and SIRs, Systemic Inflammatory Response Syndrome. High–sepsis risk bacteremia was defined as gram-negative, *Staphylococcus aureus*, or *Streptococcus* bacteremia; 10-day and 28-day mortality, death within 10 or 28 days of culture collection.

SHBSL had the highest AUROC for secondary analyses. Among location stratified cohorts, SHBSL had an AUROC of 0.82 (95% CI, 0.76-0.89) among PBIs identified in inpatient settings and 0.82 (95% CI, 0.75-0.89) among PBIs identified in outpatient settings. Inpatient PBIS had higher estimated scores than outpatient PBIs (eFigure 5 in the Supplement), and the optimal cut points for SHBSL, C-SBHSL, and NEWS varied by location (Table 2). SHBSL had an AUROC of 0.85 (95% CI, 0.79-0.91) for 10-day mortality and 0.80 (95% CI, 0.75-0.84) for 28-day mortality. When compared with the primary analysis (all PBIs), existing tools had lower outpatient AUROCs but higher short-term mortality AUROCs (Table 3 and Figure 2). SHBSL had a cross-validated AUC of 0.85 (95% CI, 0.70-0.91), and C-SHBSL had a cross-validated AUC of 0.74 (95% CI, 0.59-0.86) (eFigure 6 and eTable 6 in the Supplement). SIRS had the highest bootstrapped AUC, at 0.64 (95% CI, 0.52-0.77) (eTable 6 in the Supplement).

**Table 3.** Prognostic Value of Sepsis Prognosis Tools for High–Sepsis Risk Bacteremia Stratified by Patient Location at Time of Culture Collection and for Short-term Mortality Among Recipients of Allogeneic Hematopoietic Cell Transplant With Potential Bloodstream Infections

Measure	AUROC^a^	Sensitivity^b^	Specificity^b^	Upper-left cut point^c^
**High–sepsis risk bacteremia: Patient location stratified PBIs**				
Inpatient				
Clinical factor–specific SHBSL^d^	0.64 (0.54-0.74)	97.5 (84.9%-99.6%)	7.6 (5.4%-10.5%)	17.5%
Full SHBSL^d^	0.82 (0.76-0.89)	97.5 (84.2%-99.6%)	29.9 (24.9%-35.3%)	9.1%
SIRS (≥2)	0.57 (0.48-0.66)	67.5 (51.3%-80.4%)	53.0 (48.3%-57.7%)	2
qSOFA (≥2)	0.53 (0.46-0.60)	7.5 (2.4%-20.9%)	98.0 (96.1%-99.0%)	1
NEWS	0.60 (0.50-0.70)	NA	NA	3
Cut point of ≥4	NA	40.0 (26.3%-55.5%)	77.5 (72.9%-81.5%)	NA
Cut point of ≥7	NA	10.0 (3.8%-23.9%)	95.4 (93.0%-97.0%)	NA
Outpatient				
Clinical factor–specific SHBSL ^d^	0.60 (0.50-0.69)	23.3 (11.6%-41.3%)	84.3 (82.3%-86.1%)	5.0%
Full SHBSL^d^	0.82 (0.75-0.89)	56.7 (40.1%-71.9%)	85.4 (82.3%-87.9%)	3.8%
SIRS (≥2)	0.57 (0.46-0.68)	36.7 (21.5%-55.0%)	82.6 (80.1%-84.7%)	2
qSOFA (≥2)	0.48 (0.41-0.55)	6.7 (1.7%-22.9%)	99.9 (99.5%-100%)	1
NEWS	0.56 (0.46-0.65)	NA	NA	2
Cut point of ≥4	NA	20 (8.3%-40.8%)	83.2 (81.2%-85%)	NA
Cut point of ≥7	NA	6.7 (1.7%-22.9%)	99.6 (99.2%-99.8%)	NA
**Short-term mortality**				
10-d mortality				
Clinical factor–specific SHBSL^d^	0.76 (0.68-0.83)	80.6 (62.9%-91.1%)	66.6 (63.7%-69.3%)	10.0%
Full SHBSL^d^	0.85 (0.79-0.91)	90.3 (73.5%-96.9%)	72.0 (68.7%-75.1%)	5.0%
SIRS (≥2)	0.63 (0.53-0.73)	45.2 (26.7%-65.1%)	75.2 (73.0%-77.3%)	3
qSOFA (≥2)	0.64 (0.54-0.74)	22.6 (10.1%-43.1%)	99.6 (99.2%-99.8%)	1
NEWS	0.71 (0.61-0.81)	NA	NA	3
Cut point of ≥4	NA	41.9 (20.5%-66.9%)	81.8 (80.0%-83.5%)	NA
Cut point of ≥7	NA	16.1 (7.5%-31.3%)	98.7 (98.1%-99.1%)	NA
28-d mortality				
Clinical factor–specific SHBSL^d^	0.71 (0.66-0.77)	71.1 (59.2%-80.7%)	67.4 (64.6%-70.1%)	9.9%
Full SHBSL^d^	0.80 (0.75-0.84)	78.9 (66.3%-87.7%)	73.2 (70.0%-76.3%)	5.0%
SIRS (≥2)	0.65 (0.60-0.70)	44.4 (31.9%-57.7%)	75.7 (73.5%-77.8%)	2
qSOFA (≥2)	0.58 (0.53-0.63)	8.9 (3.9%-18.8%)	99.6 (99.2%-99.8%)	1
NEWS	0.64 (0.58-0.70)	NA	NA	3
Cut point of ≥4	NA	37.8 (26.7%-50.3%)	82.3 (80.5%-84.0%)	NA
Cut point of ≥7	NA	8.9 (4.8%-15.8%)	98.8 (98.2%-99.2%)	NA

Abbreviations: AUROC, area under the receiver operating characteristic curve; NA, not applicable; NEWS, National Early Warning Score; qSOFA, quick Sequential Organ Failure Assessment; SHBSL, super hematopoietic cell transplant bacterial sepsis learner; SIRS, Systemic Inflammatory Response Syndrome.

^a^ 95% CI estimated using 2000 stratified bootstrapped replicates.

^b^ AUROC and 95% CI estimated using generalized estimating equations with robust standard errors.

^c^ Risk-probability cut point closest to the 01 (upper left) corner of the ROC.

^d^ Based on upper-left selected cut points from primary validation analysis (clinical factor–specific SHBSL: 9.3%; SHBSL: 5.0%).

The mean estimated high-risk bacteremia probability of each tool matched the observed proportion in the validation data set (3.1%; eTable 7 in the Supplement) The calibration plots show that SHBSL had the best overall calibration (ie, the estimated vs observed probability line falls closest to the ideal [diagonal] calibration line) (Figure 1C).

### Sensitivity Analyses

SHBSL had the highest AUROC in all examined scenarios and a higher pr-AUC than existing tools under all examined scenarios (eTables 8-12 in the Supplement) and C-SHBSL for all but the scenario only including non-Hispanic White individuals (eTable 9 in the Supplement). SHBSL and C-SHBSL had lower AUROCs when the surveillance cultures were removed than in the primary analysis. Under varying culture collection (eTable 11 in the Supplement), racial/ethnic makeup (eTable 9 in the Supplement), and missingness (eTable 8 in the Supplement) scenarios, SHBSL had higher AUROCs than those of existing tools, and a primary analysis selected optimal cut point sensitivity between 70.0% and 94.1% and specificity between 72.3% and 75.8%.

## Discussion

In this prognostic study, we built 2 automated prognosis tools to provide sepsis-related treatment decision support at the time of blood culture collection among recipients of allo-HCT in inpatient or outpatient settings. We developed these tools using a state-of-the-art ensemble modeling technique (the Super Learner) and included demographic and vital factors in both tools as well as a wide array of clinical and transplant factors in SHBSL. Compared with existing clinical sepsis tools, these tools had higher estimated discrimination and calibration for high–sepsis risk bacteremia and short-term mortality among recipients of allo-HCT with PBIs.

Regardless of cohort, sepsis proxy end point, or data scenario, SHBSL had a moderate to high AUROC, and C-SHBSL had a moderate AUROC, and existing tools had low to moderate AUROCs. In addition, SHBSL had higher PPVs for all sensitivities than existing tools and relative to all sensitivities greater than 20% compared with C-SHBSL. These results suggests that the additional data cleaning and processing required to run SHBSL instead of C-SHBSL are justified, that SHBSL can be run on complete or incomplete data, the superior observed performance of SHBSL is likely generalizable to other transplant centers, SBHSL can be run in inpatient or outpatient settings (but location-specific cut points are needed), and SHBSL would likely result in fewer false flags relative to any sensitivity than existing tools. Finally, the fact that SHBSL had superior prognostic performance irrespective of sepsis-related end point suggests that, compared with existing tools, SHBSL is better able to estimate sepsis risk, not just high-risk bacteremia, among recipients of allo-HCT with PBIs.

We observed lower than expected sensitivities for NEWS and SIRS for short-term mortality. Previous 10-day mortality sensitivity estimates for NEWS at the 7 or greater cut point and SIRS at the 2 or greater cut point among recipients of allo-HCT were 62 percentage points higher for NEWS at the 7 or greater cut point and 46 percentage points higher for SIRS at the 2 or greater cut point than our estimates.^11^ This difference is likely driven by our decision to evaluate potential infections (ie, all blood cultures) instead of the commonly used suspected infections (ie, body fluid cultures collected within a specific antibiotic time epoch) as well as missingness frequency differences (suspected infection study temperature missingness, 6%; temperature missingness in this study, 35%).^11,18^ While examining PBIs likely contributed to our large amount of missingness and meant we included some cultures in the absence of clinical suspicion of infection (ie, surveillance cultures), we evaluated PBIs rather than suspected infections because our primary end point required blood culture confirmation, and we wanted our tools to inform antibiotic treatment use rather than estimate disease escalation among patients already receiving antibiotic treatment.^34,37^ To ensure our tools were not biased toward surveillance cultures, we performed a sensitivity analysis excluding surveillance cultures and found that neither C-SHBSL nor SHBSL performed meaningfully different.

Unlike most automated sepsis estimation tools, which were built to improve early detection of clinically identified sepsis through real-time estimation, we built SBHSL and C-SHBSL to improve the accuracy of the sepsis risk estimation itself. This decision was driven by the atypical presentation of sepsis within our study population population, the desire to build tools functional in both inpatient and outpatient settings (limited continuous data), the limited accuracy of existing clinical scores, and reliance on outcomes that require cultures (absence of culture may not equal negative culture). While this prevents direct comparison between SBHSL or C-SHBSL and real-time tools, the performances of real-time tools provide a reference of what may be considered good. According to a 2020 systematic review,^38^ real-time tools and SHBSL had similar AUROCs. This comparison demonstrates that not only does SHBSL have superior performance compared with existing clinical tools, its performance is numerically comparable to state-of-the-art real-time estimation tools.

### Strengths and Limitations

Our study has several strengths. First, the 100-day observation of our sample allowed us to develop bacterial sepsis prognosis tools for recipients throughout their most immunocompromised periods regardless of their inpatient status. Second, our data-driven modeling approach allowed the unique presentation of bacterial sepsis within this population to be captured. Third, we showed that our prognosis tool evaluation was robust to numerous assumptions made, including those regarding missing data and culture inclusion. Fourth, through secondary analyses using alternative end points, we were able to show that SHBSL captured risk, regardless of proxy end point, with similarly high AUROC.

Our study also has several limitations. First, our sample is reflective of the northwestern US transplant community but may not reflect the larger international transplant population. Second, our tools rely on culture collection and are potentially biased toward our center’s practices. Third, our analyses relied on retrospective data and suffered from data collection and cleaning related biases. Such limitations are not unique to our study and stem from our inability to evaluate our tools externally or prospectively at the time of this study. Although we performed numerous sensitivity analyses testing our tools’ generalizability, future analyses should validate them among prospectively collected and external data. In addition, while it is possible to estimate factor importance from the Super Learner, the inclusion of correlated factors (eg, weight and height) prevented reliable estimation.^39,40^ Furthermore, we were unable to account for potential within-person correlation in our AUROC and NRI uncertainty estimates; 95% CIs accounting for this may differ slightly from those presented. Additionally, despite rapid advancement in EMR systems, EMR integration of automated tools, including ours, would be costly and, in the absence of a well-maintained and organized system, may be impossible. Furthermore, the Super Learner ensembles had lower (<1%) cross-validated AUCs than a supplied algorithm; however, we presented the ensembles as our final models because they provide the least biased options for future updates (eAppendix 5 in the Supplement).

## Conclusions

In this prognostic study, we built 2 automated bacterial sepsis decision support tools for recipients of allo-HCT with PBIs in either inpatient or outpatient settings. When compared with existing tools and C-SHBSL, SHBSL had superior performance regardless of patient location at time of culture collection and examined sepsis-related end point. Our results suggest that, if SHBSL were to be run at the time of blood culture collection, it would provide clinically relevant information regarding bacterial sepsis risk and antibiotic initiation needs of recipients of allo-HCT.

## Notes

Supplement. **eFigure 1.** Bacterial Sepsis Prognosis Tool Schematic **eAppendix 1.** Outcome Justification **eTable 1.** Frequency of Potential Factors Screened for Model Inclusion **eFigure 2.** Missingness Frequency and Pattern for Potential Model Factors **eAppendix 2.** Imputation Approach **eTable 2.** Reference Value Ranges for Single Reference Value Imputation **eAppendix 3.** Detailed Description of Super Learner Library **eAppendix 4.** Net Reclassification **eFigure 3.** Flowchart of Hematopoietic Cell Transplant Recipient (HCT) Study Population and Potential Bloodstream Infection Cohort **eFigure 4.** Histograms of Location of Patient and Collected Cultures by Day Since Transplant **eTable 3.** Population Demographic and Transplant Factors by Randomly Assigned Modeling and Validation Dataset **eTable 4.** Discrimination and Predictive Accuracy of Sepsis Prognosis Tools for High–Sepsis Risk Bacteremia **eTable 5.** Categorical Reclassification Index Comparing Risk Classification of Full Predictor Tool (SHBSL) With Other Examined Tools **eFigure 5.** Summary of Prediction Scores by Patient Location **eAppendix 5.** Ensemble Selection Rational **eFigure 6.** Cross-Validated Area Under the Curve Estimates of Super Learner and User-Supplied Algorithms **eTable 6.** Cross-Validated and Bootstrapped Area Under the Curve Estimates for High–Sepsis Risk Bacteremia **eTable 7.** Calibration and Observed vs Estimated High–Sepsis Risk Bacteremia Probabilities **eAppendix 6.** Sensitivity Analyses **eTable 8.** Predictive Ability of Examined Prognostic Tools for High–Sepsis Risk Bacteremia Among Allogeneic HCT Recipients with PBIs Under Varying Missing Data Assumptions **eTable 9.** Predicative Ability of Sepsis Prognosis Tools Among Varying Racial/Ethnic Groups **eTable 10.** Predicative Ability of Sepsis Prognosis Tools Under Varying Factor Measurement Collection Time Windows **eTable 11.** Predictive Ability of Sepsis Prognosis Tools Under Varying Culture Collection and Potential Infection Restriction Definitions **eTable 12.** Predictive Ability of Sepsis Prognosis Tools Under Varying Recent Antibiotic Definitions **eReferences**
