# Supplementary material for: Development and Validation of a Machine Learning Model to Estimate Bacterial Sepsis Among Immunocompromised Recipients of Stem Cell Transplant
Source: JAMA Netw Open. 2021 Apr 19;4(4):e214514. doi: 10.1001/jamanetworkopen.2021.4514 (PMC8056279; doi:10.1001/jamanetworkopen.2021.4514)
Supplement: Supplement. — eFigure 1. Bacterial Sepsis Prognosis Tool Schematic eAppendix 1. Outcome Justification eTable 1. Frequency of Potential Factors Screened for Model Inclusion eFigure 2. Missingness Frequency and Pattern for Potential Model Factors eAppendix 2. Imputation Approach eTable 2. Reference Value Ranges for Single Reference Value Imputation eAppendix 3. Detailed Description of Super Learner Library eAppendix 4. Net Reclassification eFigure 3. Flowchart of Hematopoietic Cell Transplant Recipient (HCT) Study Population and Potential Bloodstream Infection Cohort eFigure 4. Histograms of Location of Patient and Collected Cultures by Day Since Transplant eTable 3. Population Demographic and Transplant Factors by Randomly Assigned Modeling and Validation Dataset eTable 4. Discrimination and Predictive Accuracy of Sepsis Prognosis Tools for High–Sepsis Risk Bacteremia eTable 5. Categorical Reclassification Index Comparing Risk Classification of Full Predictor Tool (SHBSL) With Other Examined Tools eFigure 5. Summary of Prediction Scores by Patient Location eAppendix 5. Ensemble Selection Rational eFigure 6. Cross-Validated Area Under the Curve Estimates of Super Learner and User-Supplied Algorithms eTable 6. Cross-Validated and Bootstrapped Area Under the Curve Estimates for High–Sepsis Risk Bacteremia eTable 7. Calibration and Observed vs Estimated High–Sepsis Risk Bacteremia Probabilities eAppendix 6. Sensitivity Analyses eTable 8. Predictive Ability of Examined Prognostic Tools for High–Sepsis Risk Bacteremia Among Allogeneic HCT Recipients with PBIs Under Varying Missing Data Assumptions eTable 9. Predicative Ability of Sepsis Prognosis Tools Among Varying Racial/Ethnic Groups eTable 10. Predicative Ability of Sepsis Prognosis Tools Under Varying Factor Measurement Collection Time Windows eTable 11. Predictive Ability of Sepsis Prognosis Tools Under Varying Culture Collection and Potential Infection Restriction Definitions eTable 12. Predictive Ability of Sepsis Prognosis Tool [file jamanetwopen-e214514-s001.pdf]

Supplemental Online Content

Lind ML, Mooney SJ, Carone M, et al. Development and validation of a machine learning model to estimate bacterial sepsis among immunocompromised recipients of stem cell transplant. *JAMA Netw Open*. 2021;4(4):e214514. doi:10.1001/jamanetworkopen.2021.4514

eFigure 1. Bacterial Sepsis Prognosis Tool Schematic
eAppendix 1. Outcome Justification
eTable 1. Frequency of Potential Factors Screened for Model Inclusion
eFigure 2. Missingness Frequency and Pattern for Potential Model Factors
eAppendix 2. Imputation Approach
eTable 2. Reference Value Ranges for Single Reference Value Imputation
eAppendix 3. Detailed Description of Super Learner Library
eAppendix 4. Net Reclassification
eFigure 3. Flowchart of Hematopoietic Cell Transplant Recipient (HCT) Study Population and Potential Bloodstream Infection Cohort
eFigure 4. Histograms of Location of Patient and Collected Cultures by Day Since Transplant
eTable 3. Population Demographic and Transplant Factors by Randomly Assigned Modeling and Validation Dataset
eTable 4. Discrimination and Predictive Accuracy of Sepsis Prognosis Tools for High–Sepsis Risk Bacteremia
eTable 5. Categorical Reclassification Index Comparing Risk Classification of Full Predictor Tool (SHBSL) With Other Examined Tools
eFigure 5. Summary of Prediction Scores by Patient Location
eAppendix 5. Ensemble Selection Rational
eFigure 6. Cross-Validated Area Under the Curve Estimates of Super Learner and User-Supplied Algorithms
eTable 6. Cross-Validated and Bootstrapped Area Under the Curve Estimates for High–Sepsis Risk Bacteremia
eTable 7. Calibration and Observed vs Estimated High–Sepsis Risk Bacteremia Probabilities
eAppendix 6. Sensitivity Analyses
eTable 8. Predictive Ability of Examined Prognostic Tools for High–Sepsis Risk Bacteremia Among Allogeneic HCT Recipients with PBIs Under Varying Missing Data Assumptions
eTable 9. Predicative Ability of Sepsis Prognosis Tools Among Varying Racial/Ethnic Groups
eTable 10. Predicative Ability of Sepsis Prognosis Tools Under Varying Factor Measurement Collection Time Windows
eTable 11. Predictive Ability of Sepsis Prognosis Tools Under Varying Culture Collection and Potential Infection Restriction Definitions

© 2021 Lind ML et al. *JAMA Network Open.*

eTable 12. Predictive Ability of Sepsis Prognosis Tools Under Varying Recent Antibiotic Definitions
eReferences

This supplemental material has been provided by the authors to give readers additional information about their work.

© 2021 Lind ML et al. *JAMA Network Open.*

eFigure 1. Bacterial Sepsis Prognosis Tool Schematic

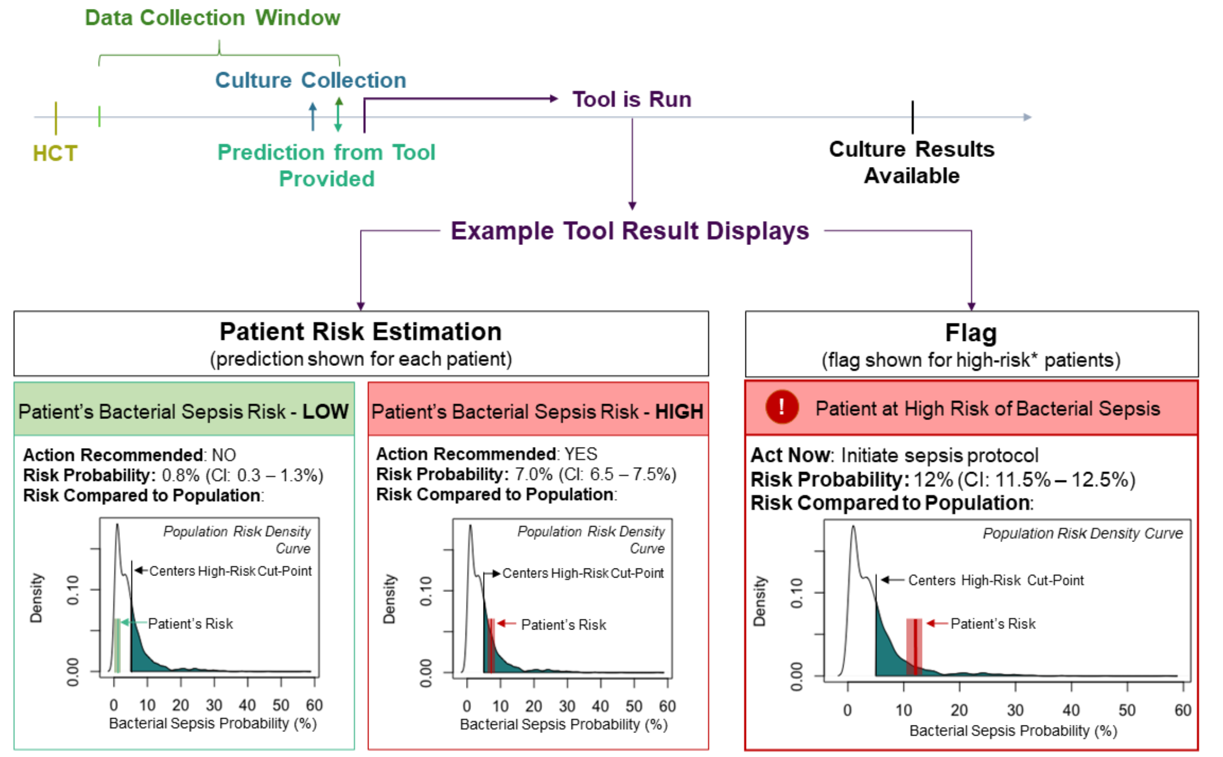

* High Risk: Minimum probability at which the sensitivity meets a health system's selected minimum allowable capture (ex. High: 90% sensitivity)

© 2021 Lind ML et al. *JAMA Network Open.*

eAppendix 1. Outcome Justification

We selected bacteremia with high risk for sepsis - blood culture with gram-negative species, *Staphylococcus aureus*, or *Streptococcus* species bacteremia – based on evidence that sepsis is significantly more likely among HCT recipients with PBIs and the selected bloodstream infections than without. Neutropenic HCT recipients with positive blood cultures have been found to be 9.6 times and recipients with gram-negative bacteremia 12.9 times more likely to have severe sepsis than individuals with negative blood cultures.[1] Additionally, previous studies show gram-negative bacteremia results in significantly higher C-reactive protein levels (a recognized indicator of sepsis[2]) and is associated with a significantly higher risk of severe sepsis and non-relapse mortality than gram-positive bacteremia.[1,3]

© 2021 Lind ML et al. *JAMA Network Open.*

eTable 1. Frequency of Potential Factors Screened for Model Inclusion

Factor	Considered in:	Included in:	Missing	Range	Median/ Most Frequent Category	IQR/Percent of Most Frequent
**Demographic**						
Age	C-SHBSL/SHBSL	C-SHBSL/SHBSL	0, 0%	18, 80	53.1	40.6, 61.9
Ethnicity/Race	C-SHBSL/SHBSL	C-SHBSL/SHBSL	0, 0%	-	White	78.8%
Gender	C-SHBSL/SHBSL	C-SHBSL/SHBSL	0, 0%	-	Male	57.5%
**Body Structure**						
BMI at time of transplant	C-SHBSL/SHBSL	C-SHBSL/SHBSL	0, 0%	12.8, 44	24.7	21.7, 28.1
**Vitals and Biomarkers**						
Albumin	SHBSL	SHBSL	923, 11.4%	1, 4.9	3.5	3.1, 3.8
Alanine Aminotransferase	SHBSL	SHBSL	1444, 17.8%	3, 9380	26	16, 46
Antithrombin III	SHBSL		8130, 100%	136, 136	136	136, 136
Asparate Transaminase	SHBSL	SHBSL	1441, 17.7%	4, 11290	21	16, 29
Bicarbonate	SHBSL		8131, 100%	-	-	-
Bilirubin	SHBSL	SHBSL	1442, 17.7%	0.1, 26.5	0.7	0.5, 1
Blood Urea Nitrogen	SHBSL	SHBSL	591, 7.3%	1, 159	22	16, 29

© 2021 Lind ML et al. *JAMA Network Open.*

C-Reactive Protein	SHBSL		8131, 100%	-	-	-
Creatinine	SHBSL	SHBSL	589, 7.2%	0.2, 8.9	1	0.8, 1.2
Diastolic Blood Pressure	C-SHBSL/SHBSL	C-SHBSL/SHBSL	2465, 30.3%	13, 134	79	72, 86
Factor IX	SHBSL		8130, 100%	90, 90	90	90, 90
Factor V	SHBSL		8130, 100%	38, 38	38	38, 38
Glascow Coma Score	C-SHBSL/SHBSL		7497, 92.2%	4, 15	15	15, 15
Glucose	SHBSL	SHBSL	592, 7.3%	54, 556	132	111, 164
Heart Rate (Pulse)	C-SHBSL/SHBSL	C-SHBSL/SHBSL	2266, 27.9%	11, 170	90	79, 103
Hemoglobin	SHBSL	SHBSL	463, 5.7%	5.7, 16	10.2	9.4, 11.3
International Normalized Ratio	SHBSL		8131, 100%	-	-	-
Interleukin-6	SHBSL		8131, 100%	-	-	-
Lactate (volumn) within last 24 hours	SHBSL		7962, 97.9%	0.3, 11.8	1.3	0.9, 2.1
Lactate Dehydrogenase	SHBSL		8004, 98.4%	65, 1495	51	25.5, 78.5
Neutrophil	SHBSL	SHBSL	759, 9.3%	0, 43	3.8	1.9, 6.5
Oxygen Saturation	C-SHBSL/SHBSL		5153, 63.4%	84, 100	97	95, 98
Plasminogen activator inhibitor 1	SHBSL		8131, 100%	-	-	-
Plasminogen activity	SHBSL		8131, 100%	-	-	-
Platelet	SHBSL	SHBSL	430, 5.3%	1, 745	88	46, 145
Protein C Activity	SHBSL		8125, 99.9%	167, 242	201	195.8, 212.2
Protein S Activity	SHBSL		8131, 100%	-	-	-

© 2021 Lind ML et al. *JAMA Network Open.*

Prothrombin	SHBSL		7480, 92%	0.8, 2.5	1.1	1, 1.2
Respiratory Rate	C-SHBSL/SHBSL	C-SHBSL/SHBSL	2375, 29.2%	6, 56	16	16, 18
Sodium	SHBSL	SHBSL	587, 7.2%	117, 152	136	134, 138
Supplemental Oxygen (Y/N)	C-SHBSL/SHBSL	C-SHBSL/SHBSL	0, 0%	0, 100	0	0, 0
Systolic Blood Pressure	C-SHBSL/SHBSL	C-SHBSL/SHBSL	2464, 30.3%	73, 206	128	116, 139.5
**Factor**	**Considered in:**	**Included in:**	**Missing**	**Range**	**Median/ Most Frequent Category**	**IQR/Percent of Most Frequent**
Temperature	C-SHBSL/SHBSL	C-SHBSL/SHBSL	2698, 33.2%	34.5, 40.1	36.9	36.6, 37.3
White Blood Cell Count	SHBSL	SHBSL	919, 11.3%	0.1, 19.9	5	2.8, 8.1
**Recent Events**						
Previous Bacterial Sepsis Event	SHBSL	SHBSL	0, 0%	0, 4	0	0, 0
Antibiotics within last 14 days	SHBSL	SHBSL	0, 0%	0, 1	1	0, 1
Days Since Transplant	SHBSL	SHBSL	0, 0%	0, 100	54	34, 74
Time of Day (in hours 0-24)	SHBSL	SHBSL	0, 0%	0, 24	10	9, 12
**Additional Factors**						
Location (IUC, inpatient, v outpatient)	SHBSL	SHBSL	0, 0%	-	Outpatient	74.4%
Acute GVHD[a]	SHBSL	SHBSL	0, 0%	0, 4	2	2, 2
Acute Gut GVHD[a]	SHBSL	SHBSL	0, 0%	0, 4	1	1, 1
Acute Skin GVHD[a]	SHBSL	SHBSL	0, 0%	0, 4	1	0, 3
Acute Liver GVHD[a]	SHBSL	SHBSL	0, 0%	0, 4	0	0, 0

© 2021 Lind ML et al. *JAMA Network Open.*

Donor Relation (related/unrelated)	SHBSL	SHBSL	0, 0%	-	Not Related	69.9%
HCT Cell Donor Type (ex. BM/PBSC)	SHBSL	SHBSL	0, 0%	-	Unrelated	69.9%
Underlying disease/cancer type	SHBSL	SHBSL	0, 0%	-	ANL	33.2%
Disease Status	SHBSL	SHBSL	0, 0%	-	Remission	54.8%
[a]GVHD: Graft vs. Host Disease						

© 2021 Lind ML et al. *JAMA Network Open.*

eFigure 2. Missingness Frequency and Pattern for Potential Model Factors

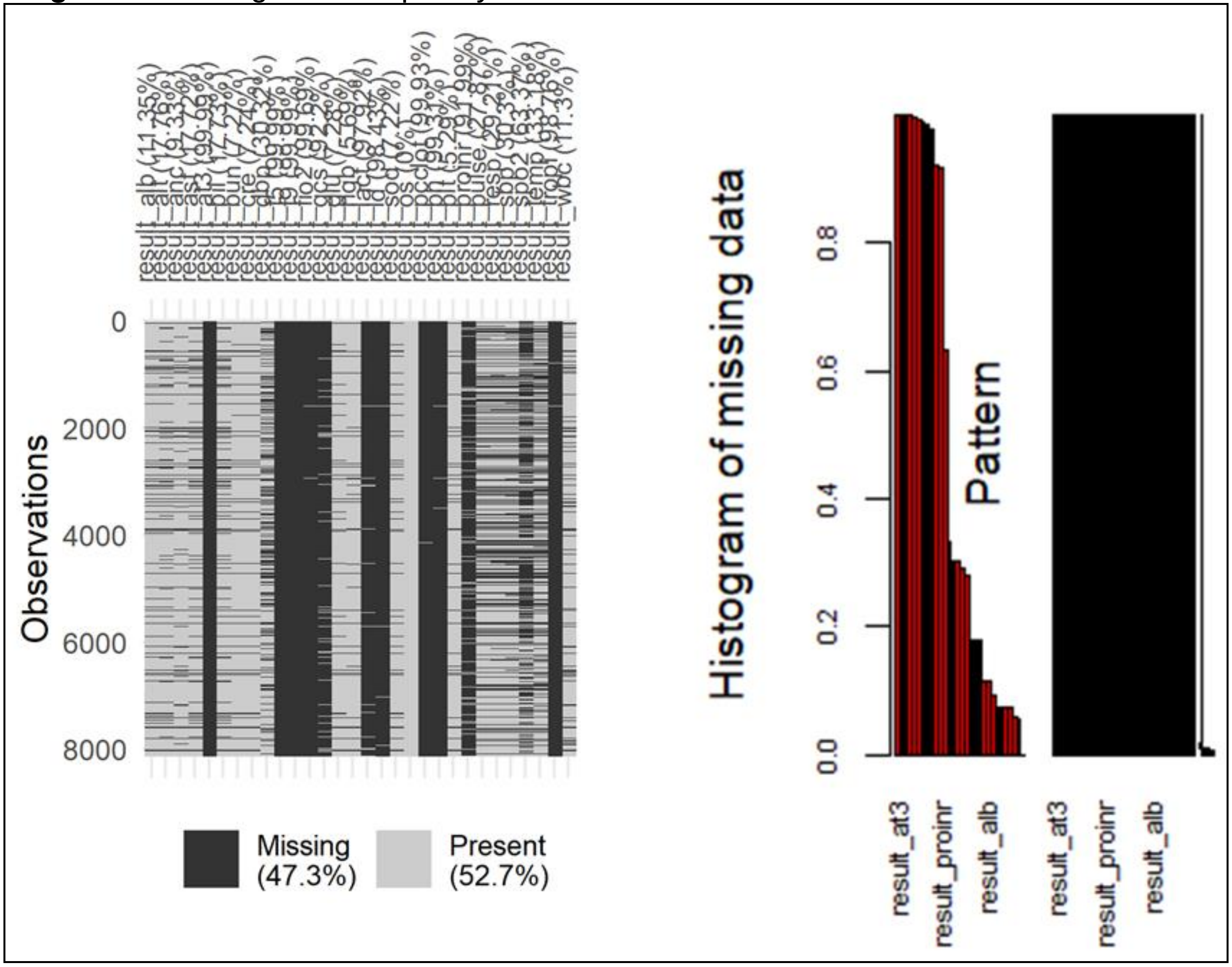

© 2021 Lind ML et al. *JAMA Network Open.*

eAppendix 2. Imputation Approach

Given this data limitation, we employed a two-step missing data approach. First, we restricted to factors that had measurements for at least 65% of the examined PBIs. Second, we filled the remaining missing measurement observations using a single, normal value imputation approach. Specially for each missing value, we randomly selected a value from the normal biological range of that factor. The specific normal value rangers used can be found in are e.Table2.

© 2021 Lind ML et al. *JAMA Network Open.*

eTable 2. Reference Value Ranges for Single Reference Value Imputation

	Normal Value Range	Source
Temperature	36.1 - 37.2°C	MedlinePlus
Respiratory Rate	12-20 breaths/minute	Cleveland Clinic
Pulse (Heart Rate)	60-100 beats/minute	Mayo Clinic
Systolic Blood Pressure	90-120 mmHg	Havard, Tolulope et al.
Diastolic Blood Pressure	60-80 mmHg	Stensrud et al.
Oxygen Saturation	95-100%	Mayo Clinic
White Blood Cell Count	4.4 - 11 billion cells/L	UpToDate
Albumin	3.4 - 5.4 g/dL	Rochester
Alanine Aminotransaminase	7 - 55 u/l	Mayo Clinic
Asparate Transaminase	8 - 48 u/l	Mayo Clinic
Bilirubin	0.1 - 1.2 mg/dL	Mayo Clinic
Blood Urea Nitrogen	7 - 20 mg/dL	Mayo Clinic
Creatinine	0.84 - 1.21 mg/dL	Mayo Clinic
Glucose	62 - 125 mg/dL	Mindscape
Sodium	135 - 145 mEq/L	Mayo Clinic
Absolute Neutrophil Count	1.5 - 8 n/nL	Healthline
Platelet	150 - 450 p/nL	Hopkins Medicine
Hemoglobin		Mayo Clinic
Female	12- 15.5 g/dL	
Male	13.5 - 17.5 g/dL	
Glasgow Coma Scale Score[a]	15	MdCalc
[a] For existing criteria comparisons		

© 2021 Lind ML et al. *JAMA Network Open.*

eAppendix 3. Detailed Description of Super Learner Library

Screens: predictor selection step that can be applied to included algorithms thus reducing the predictors included in algorithm learning

- Correlation pvalue (corP): predictors with a pearson correlation pvalue of 0.05 or less are included (user edited)
- Regularization (glmnet): performs L1 (Lasso) penalized regression and keeps predictors with coefficients larger than zero (original)

Algorithms: varying algorithms and algorithm forms (screened and hypermeters)

- Linear Models
    - Logistic regression: SL.glm
        - Base
        - Screen: corP & glmnet
    - Stepwise regression: SL.stepAIC
        - Base
    - Generalized additive model: SL.gam
        - Base
        - Screen: corP & glmnet
    - Penalized generalized linear model using elastic net: SL.glmnet
        - Base: regularization L1 (alpha 1)
        - Hyperparameter
            - Regularization: L2 (alpha 0)
        - Screen: corP
- Network and Trees
    - Single Node Neural Network: SL.nnet
        - Hyperparameters: Decays (regularization): 0.9, Size (number of units in hidden layer): 6
            - Screen: corP
    - Recursive Binary Split Tree (Classification): SL.rpart
        - Base
    - Random Forest (classification tree): SL.randomForest
        - Base
        - Screen: corP & glmnet
        - Hyperparameters: group size of 100
            - Screen: corP & glmnet
    - Bagging classification tree: SL.ipredbagg
        - Base
        - Screen: corP

The parameters used in the base form of each algorithm can be found in Dr. Eric Polley's Github SuperLearner R code repository.

© 2021 Lind ML et al. *JAMA Network Open.*

eAppendix 4. Net Reclassification

Net Reclassification Index (NRI) is a measure of tool discrimination that works by comparing how patients distributed into risk probability categories by two models (say SIRS and SHBSL) actually fare (in relation to a specific outcome – such as high-risk bacteremia). The NRI conducts this comparison by measuring the net improvement of appropriate patient placement (i.e. events in higher prediction categories) of one model (say SHBSL) over the other (say SIRS).

To calculate this metric, we considered SHBSL to be the "updated" model and the existing tool or C-SHBSL to be the "base" model. In order to get risk probability categories for existing tools, we estimated the risk probabilities associated with each score for SIRS, NEWS, and qSOFA using single variable logistic regressions. Using the resulting risk probability categories, we estimated the reclassification of SHBSL in reference to the existing tools. In order to calculate the NRI between SHBSL and C-SHBSL, we defined our categories as the quartiles of C-SHBSL's risk probabilities.

© 2021 Lind ML et al. *JAMA Network Open.*

eFigure 3. Flowchart of Hematopoietic Cell Transplant Recipient (HCT) Study Population and Potential Bloodstream Infection Cohort

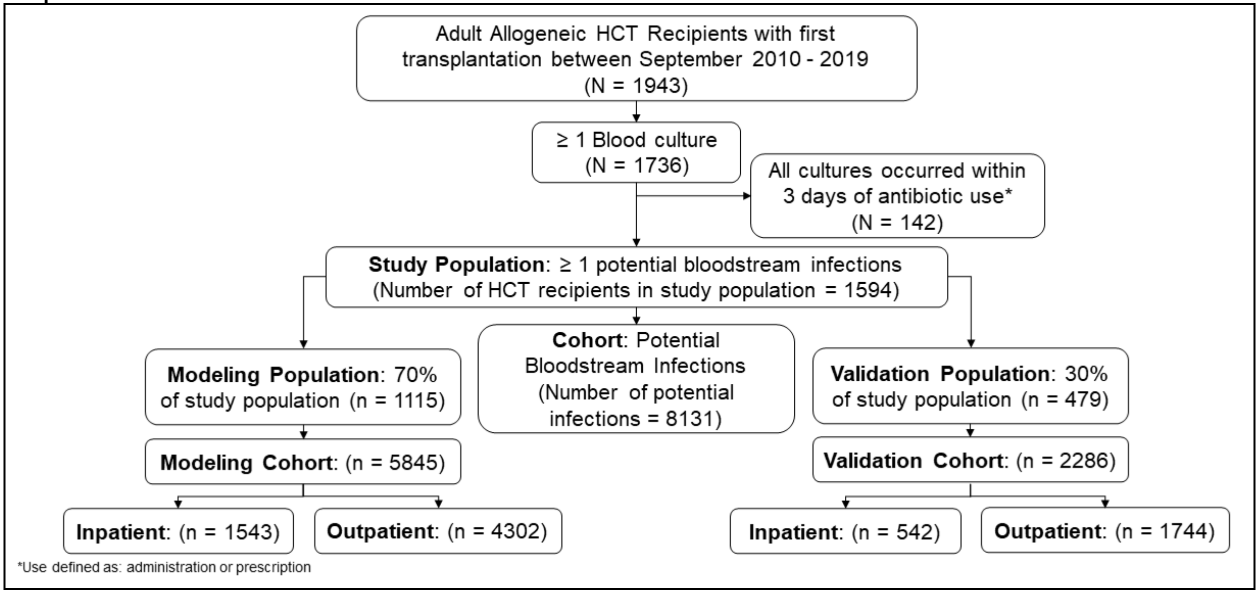

© 2021 Lind ML et al. *JAMA Network Open.*

eFigure 4. Histograms of Location of Patient and Collected Cultures by Day Since Transplant

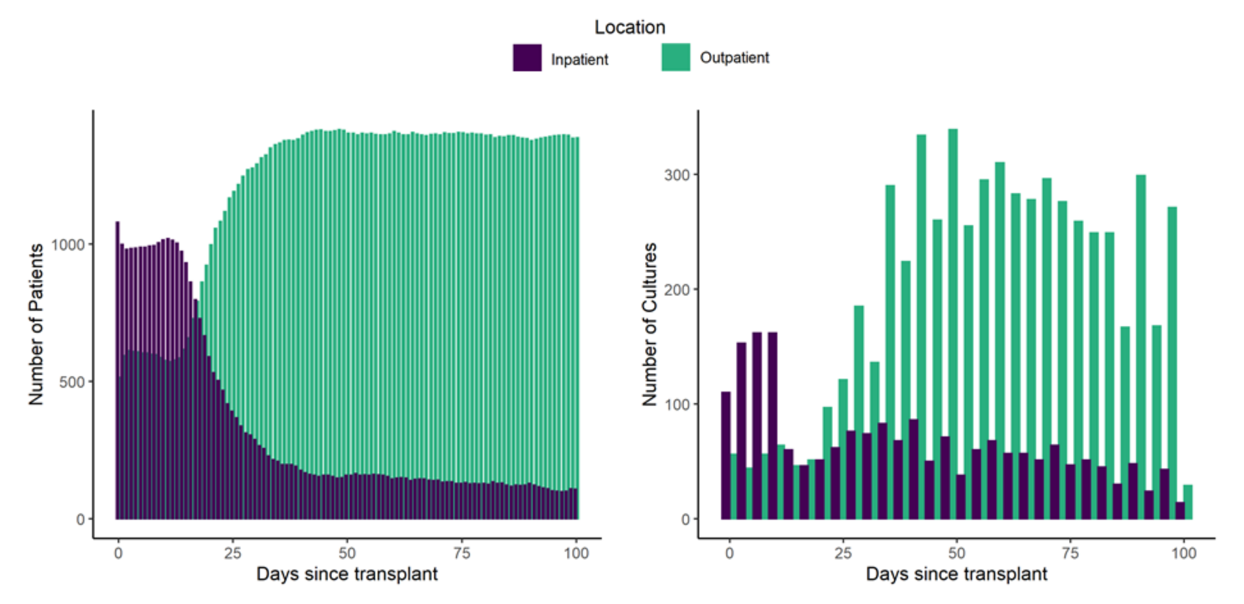

Patient location was defined using full calendar days and patients were considered to be inpatient for the full day of hospital admission and discharge. Culture location was defined based on the patient location for each day.

© 2021 Lind ML et al. *JAMA Network Open.*

eTable 3. Population Demographic and Transplant Factors by Randomly Assigned Modeling and Validation Dataset

	**Modeling**	**Validation**
	N = 1115	N = 479
Calendar Year at Tx (Median, IQR)	2014 (2012, 2016)	2014 (2012, 2016)
Age at Tx (Median, IQR)	55 (43, 63)	54 (41, 62)
Gender (Count, % male)	644 (57.8)	267 (55.7)
Ethnicity/Race (Count., %)		
American Indian or Alaskan Native[b]	19 (1.7)	5 (1.0)
Asian[b]	73 (6.5)	46 (9.6)
Black[b]	18 (1.6)	7 (1.5)
Multiple[b]	16 (1.4)	6 (1.3)
Native Hawaiian or other Pacific Islander[b]	20 (1.8)	5 (1.0)
Unknown[b]	35 (3.1)	19 (4.0)
White[b]	888 (79.6)	354 (73.9)
Hispanic/Latino	46 (4.1)	37 (7.7)
Underlying Disease		
Acute Lymphoblastic Leukemia	156 (14.0)	56 (11.7)
Acute Myelogenous Leukemia	404 (36.2)	176 (36.7)
Myelodysplastic Syndromes	297 (26.6)	113 (23.6)
Other	258 (23.1)	134 (28.0)
HCT Cell Donor Type		
Bone marrow	90 (8.1)	36 (7.5)
Bone marrow, peripheral blood stem cells	3 (0.3)	0 (0)
Umbilical cord blood	123 (11.0)	56 (11.7)
Peripheral blood stem cells	899 (80.6)	387 (80.8)
Donor Relation (Count, %)		
Child	22 (2.0)	16 (3.3)
Half Sibling	3 (0.3)	2 (0.4)
Not Related	781 (70.0)	326 (68.1)
Parent	11 (1.0)	6 (1.3)
Relative	1 (0.1)	0 (0)
Sibling	297 (26.6)	129 (26.9)
Acute GVHD (Count, %)		
Grade 0	238 (21.3)	90 (18.8)
Grade 1	57 (5.1)	21 (4.4)
Grade 2	649 (58.2)	303 (63.3)
Grade 3	129 (11.6)	50 (10.4)
Grade 4	42 (3.8)	15 (3.1)
Died (n, %)[a]	96 (8.6)	42 (8.8)

[a] Experiences during follow-up

[b] Non-Hispanic

© 2021 Lind ML et al. *JAMA Network Open.*

eTable 4. Discrimination and Predictive Accuracy of Sepsis Prognosis Tools for High–Sepsis Risk Bacteremia[a]

	Cut Points	Sensitivity[a]	Specificity[a]	Positive Predictive Value[a]	Negative Predictive Value[a]
Clinical Factor Specific - C-SHBSL					
	0.0214	100 (100, 100%)	1.7 (1.2, 2.4%)	3.1 (2.3, 4.1%)	100 (100, 100%)
	0.0310	98.6 (91.2, 99.8%)	9.9 (8.7, 11.3%)	3.3 (2.5, 4.4%)	99.5 (96.9, 99.9%)
	0.0407	97.1 (90.0, 99.2%)	21.7 (19.8, 23.8%)	3.8 (2.9, 5.0%)	99.6 (98.4, 99.9%)
	0.0436	95.7 (84.3, 98.9%)	24.4 (22.4, 26.6%)	3.8 (2.9, 5.0.%)	99.4 (97.7, 99.9%)
	0.0439	94.3 (83.6, 98.2%)	24.8 (22.8, 27.0%)	3.8 (2.9, 5.0%)	99.3 (97.6, 99.8%)
	0.0439	92.9 (82.6, 97.3%)	24.9 (22.9, 27.1%)	3.8 (2.9, 4.9%)	99.1 (97.5, 99.7%)
	0.0450	91.4 (81.2, 96.3%)	26.3 (24.1, 28.5%)	3.8 (2.9, 5.0%)	99.0 (97.5, 99.6%)
	0.0497	90.0 (79.9, 95.3%)	31.5 (29.2, 34.0%)	4.0 (3.0, 5.2%)	99.0 (97.7, 99.6%)
	0.0502	88.6 (78.4, 94.3%)	31.9 (29.5, 34.4%)	3.9 (3.0, 5.2%)	98.9 (97.6, 99.5%)
	0.0506	87.1 (76.8, 93.3%)	32.3 (29.9, 34.7%)	3.9 (2.9, 5.2%)	98.8 (97.5, 99.4%)
	0.0522	85.7 (75.6, 92.1%)	34.0 (31.6, 36.5%)	3.9 (3.0, 5.2%)	98.7 (97.4, 99.3%)
	0.0531	84.3 (74.3, 90.9%)	34.8 (32.4, 37.4%)	3.9 (3.0, 5.2%)	98.6 (97.4, 99.3%)
	0.0548	82.9 (73.0, 89.6%)	36.6 (34.1, 39.2%)	4.0 (3.0, 5.2%)	98.5 (97.4, 99.2%)
	0.0580	81.4 (71.9, 88.3%)	40.0 (37.4, 42.6%)	4.1 (3.1, 5.4%)	98.6 (97.4, 99.2%)
	0.0582	80.0 (69.4, 87.6%)	40.3 (37.7, 42.9%)	4.1 (3.1, 5.3%)	98.5 (97.2, 99.1%)
	0.0589	78.6 (67.9, 86.4%)	41.0 (38.4, 43.6%)	4.0 (3.0, 5.3%)	98.4 (97.2, 99.1%)
	0.0651	77.1 (65.9, 85.5%)	47.1 (44.4, 49.8%)	4.4 (3.3, 5.8%)	98.5 (97.3, 99.1%)
	0.0741	75.7 (64.5, 84.3%)	54.3 (51.4, 57.2%)	5.0 (3.8, 6.6%)	98.6 (97.6, 99.2%)
	0.0787	74.3 (62.4, 83.4%)	57.4 (54.5, 60.3%)	5.2 (3.9, 6.9%)	98.6 (97.6, 99.2%)

© 2021 Lind ML et al. *JAMA Network Open.*

	0.0823	72.9 (61.0, 82.2%)	59.9 (57.0, 62.8%)	5.4 (4.1, 7.2%)	98.6 (97.6, 99.2%)
	0.0836	71.4 (59.2, 81.2%)	61.2 (58.3, 64.1%)	5.5 (4.1, 7.3%)	98.5 (97.5, 99.1%)
	0.0843	70.0 (57.0, 80.4%)	61.6 (58.6, 64.5%)	5.4 (4.1, 7.2%)	98.5 (97.4, 99.1%)
	0.0853	68.6 (54.2, 80.1%)	62.5 (59.5, 65.4%)	5.5 (4.1, 7.3%)	98.4 (97.3, 99.1%)
	0.0896	67.1 (53.1, 78.7%)	65.0 (62.1, 67.9%)	5.7 (4.3, 7.6%)	98.4 (97.3, 99.1%)
	0.0926	65.7 (51.9, 77.3%)	66.9 (64.0, 69.7%)	5.9 (4.4, 7.9%)	98.4 (97.3, 99.1%)
	0.0950	64.3 (50.2, 76.3%)	68.3 (65.4, 71.1%)	6.0 (4.5, 8.1%)	98.4 (97.3, 99.0%)
	0.0961	62.9 (48.1, 75.5%)	68.7 (65.8, 71.4%)	6.0 (4.4, 8.0%)	98.3 (97.1, 99.0%)
	0.0985	61.4 (47.1, 74.0%)	69.4 (66.6, 72.2%)	6.0 (4.4, 8.0%)	98.3 (97.1, 99.0%)
	0.1001	60.0 (46.1, 72.4%)	70.4 (67.6, 73.1%)	6.0 (4.5, 8.1%)	98.2 (97.1, 98.9%)
	0.1044	58.6 (44.9, 71.1%)	73.0 (70.3, 75.5%)	6.4 (4.7, 8.6%)	98.2 (97.1, 98.9%)
	0.1081	57.1 (43.6, 69.7%)	74.7 (72.1, 77.2%)	6.7 (4.9, 9.0%)	98.2 (97.1, 98.9%)
	0.1110	55.7 (42.4, 68.2%)	76.0 (73.4, 78.4%)	6.8 (5.0, 9.2%)	98.2 (97.1, 98.9%)
	0.1120	54.3 (41.2, 66.8%)	76.6 (74.0, 79.0%)	6.8 (5.0, 9.2%)	98.1 (97.1, 98.8%)
	0.1146	52.9 (39.9, 65.4%)	77.8 (75.2, 80.1%)	7.0 (5.1, 9.5%)	98.1 (97.1, 98.8%)
	0.1223	51.4 (38.7, 64.0%)	80.7 (78.4, 82.9%)	7.8 (5.7, 10.6%)	98.1 (97.1, 98.8%)
	0.1223	50.0 (37.4, 62.6%)	80.8 (78.5, 82.9%)	7.6 (5.5, 10.4%)	98.1 (97.1, 98.8%)
	0.1227	48.6 (36.5, 60.8%)	81.0 (78.6, 83.1%)	7.5 (5.4, 10.2%)	98.0 (97.0, 98.7%)
	0.1251	47.1 (35.3, 59.3%)	81.6 (79.3, 83.7%)	7.5 (5.4, 10.3%)	98.0 (97.0, 98.7%)
	0.1284	45.7 (34, 57.9%)	82.7 (80.5, 84.7%)	7.7 (5.5, 10.7%)	98.0 (97.0, 98.6%)
	0.1297	44.3 (32.3, 57.0%)	83.2 (81.0, 85.2%)	7.7 (5.5, 10.7%)	97.9 (96.9, 98.6%)
	0.1352	42.9 (31.1, 55.5%)	84.7 (82.6, 86.7%)	8.2 (5.7, 11.4%)	97.9 (96.9, 98.6%)

© 2021 Lind ML et al. *JAMA Network Open.*

	Cut Points	Sensitivity	Specificity	Positive Predictive Value	Negative Predictive Value
	0.1513	41.4 (29.9, 54.0%)	88.8 (87.0, 90.3%)	10.4 (7.4, 14.6%)	98.0 (97.0, 98.6%)
	0.1590	40.0 (28.7, 52.5%)	90.5 (89.0, 91.9%)	11.8 (8.2, 16.5%)	97.9 (97.0, 98.6%)
	0.1743	38.6 (27.5, 51.0%)	93.0 (91.7, 94.0%)	14.8 (10.3, 20.7%)	98.0 (97.0, 98.6%)
	0.1759	37.1 (26.3, 49.5%)	93.2 (92.0, 94.2%)	14.7 (10.2, 20.7%)	97.9 (97.0, 98.6%)
	0.1780	35.7 (24.9, 48.2%)	93.6 (92.4, 94.6%)	15.0 (10.3, 21.2%)	97.9 (96.9, 98.5%)
	0.1787	34.3 (23.7, 46.7%)	93.7 (92.6, 94.7%)	14.7 (10.1, 21.1%)	97.8 (96.9, 98.5%)
	0.1799	32.9 (22.6, 45.1%)	93.9 (92.8, 94.9%)	14.6 (9.8, 21.1%)	97.8 (96.8, 98.5%)
	0.1857	31.4 (21.4, 43.6%)	94.7 (93.6, 95.6%)	15.7 (10.5, 22.9%)	97.8 (96.8, 98.4%)
	0.1877	30.0 (20.2, 42.0%)	94.9 (93.9, 95.8%)	15.7 (10.4, 23.0%)	97.7 (96.8, 98.4%)
	0.1981	28.6 (19.1, 40.4%)	96.5 (95.6, 97.2%)	20.4 (13.5, 29.7%)	97.7 (96.8, 98.4%)
	0.1996	27.1 (17.9, 38.9%)	96.6 (95.7, 97.3%)	20.0 (13.0, 29.4%)	97.7 (96.7, 98.3%)
	0.2006	25.7 (16.8, 37.3%)	96.7 (95.8, 97.4%)	19.6 (12.6, 29.1%)	97.6 (96.7, 98.3%)
	0.2012	24.3 (15.5, 35.9%)	96.8 (95.9, 97.5%)	19.3 (12.3, 29.0%)	97.6 (96.6, 98.3%)
	0.2030	22.9 (14.4, 34.3%)	96.9 (96.1, 97.6%)	19.0 (11.9, 29.0%)	97.5 (96.6, 98.2%)
	0.2088	21.4 (13.2, 32.9%)	97.5 (96.7, 98.1%)	21.1 (13.1, 32.2%)	97.5 (96.6, 98.2%)
	0.2093	20.0 (12.1, 31.3%)	97.5 (96.7, 98.1%)	20.0 (12.2, 31.1%)	97.5 (96.5, 98.2%)
	0.2155	18.6 (11.0, 29.6%)	97.9 (97.2, 98.5%)	22.0 (13.2, 34.4%)	97.4 (96.5, 98.1%)
	0.2208	17.1 (9.9, 27.9%)	98.3 (97.6, 98.8%)	24.0 (14.1, 37.7%)	97.4 (96.5, 98.1%)
	0.2208	15.7 (8.8, 26.5%)	98.3 (97.6, 98.8%)	22.4 (12.9, 36.2%)	97.4 (96.4, 98.1%)
	0.2220	14.3 (7.8, 24.8%)	98.4 (97.7, 98.9%)	21.7 (12.1, 35.9%)	97.3 (96.4, 98.0%)
	0.2237	12.9 (6.7, 23.2%)	98.5 (97.8, 98.9%)	20.9 (11.3, 35.6%)	97.3 (96.3, 98.0%)

© 2021 Lind ML et al. *JAMA Network Open.*

	Cut Points	Sensitivity	Specificity	Positive Predictive Value	Negative Predictive Value
	0.2404	11.4 (5.7, 21.5%)	99.5 (99.0, 99.7%)	40.0 (20.9, 62.7%)	97.3 (96.3, 98.0%)
	0.2443	10.0 (4.8, 19.7%)	99.6 (99.2, 99.8%)	43.7 (21.7, 68.6%)	97.2 (96.3, 97.9%)
	0.2471	8.6 (3.9, 17.9%)	99.6 (99.2, 99.8%)	42.9 (19.8, 69.6%)	97.2 (96.2, 97.9%)
	0.2499	7.1 (3.0, 16.1%)	99.7 (99.3, 99.9%)	41.7 (17.5, 70.6%)	97.1 (96.2, 97.9%)
	0.2527	5.7 (2.2, 14.3%)	99.7 (99.3, 99.9%)	36.4 (13.6, 67.5%)	97.1 (96.1, 97.8%)
	0.2647	4.3 (1.4, 12.4%)	99.9 (99.6, 100%)	50.0 (16.8, 83.2%)	97.1 (96.1, 97.8%)
	0.2807	2.9 (0.7, 10.6%)	100 (99.7, 100%)	66.7 (15.4, 95.7%)	97.0 (96.0, 97.8%)
	0.2890	1.4 (0.2, 9.3%)	100 (99.7, 100%)	50.0 (5.9, 94.1%)	97.0 (96.0, 97.7%)
	0.3014	0.0 (0.0, 0.0%)	100 (99.7, 100%)	0.0 (0.0, 0.0%)	96.9 (95.9, 97.7%)
Full Predictor-SHBSL					
	0.0057	100 (100, 100%)	9.9 (8.0, 12.2%)	3.4 (2.6, 4.5%)	100 (100, 100%)
	0.0130	98.6 (90.5, 99.8%)	29.8 (26, 34%)	4.2 (3.2, 5.6%)	99.8 (98.9, 100%)
	0.0194	97.1 (89.4, 99.3%)	37.1 (33.0, 41.4%)	4.7 (3.5, 6.1%)	99.8 (99.0, 99.9%)
	0.0194	95.7 (87.9, 98.6%)	37.1 (33.0, 41.4%)	4.6 (3.5, 6.0%)	99.6 (98.9, 99.9%)
	0.0256	94.3 (86.3, 97.7%)	45.2 (41.1, 49.4%)	5.2 (3.9, 6.8%)	99.6 (98.9, 99.9%)
	0.0362	92.9 (84.4, 96.9%)	60.4 (56.7, 64.0%)	6.9 (5.2, 9.0%)	99.6 (99.1, 99.8%)
	0.0379	91.4 (82.8, 95.9%)	62.6 (59.0, 66.1%)	7.2 (5.5, 9.4%)	99.6 (99.0, 99.8%)
	0.0392	90.0 (81.7, 94.8%)	64.0 (60.4, 67.4%)	7.3 (5.6, 9.5%)	99.5 (99.0, 99.8%)
	0.0396	88.6 (78.4, 94.3%)	64.4 (60.8, 67.8%)	7.3 (5.6, 9.5%)	99.4 (98.8, 99.7%)
	0.0410	87.1 (77.2, 93.1%)	65.3 (61.8, 68.8%)	7.4 (5.6, 9.5%)	99.4 (98.7, 99.7%)
	0.0440	85.7 (75.9, 92.0%)	68.5 (65.1, 71.8%)	7.9 (6.1, 10.2%)	99.3 (98.7, 99.7%)

© 2021 Lind ML et al. *JAMA Network Open.*

	Cut Points	Sensitivity	Specificity	Positive Predictive Value	Negative Predictive Value
	0.0442	84.3 (74.3, 90.9%)	68.6 (65.1, 71.9%)	7.8 (6.0, 10.1%)	99.3 (98.6, 99.6%)
	0.0449	82.9 (73.0, 89.6%)	69.1 (65.6, 72.3%)	7.8 (6.0, 10.1%)	99.2 (98.6, 99.6%)
	0.0468	81.4 (70.7, 88.9%)	70.5 (67.2, 73.7%)	8.0 (6.2, 10.4%)	99.2 (98.5, 99.6%)
	0.0498	80.0 (69.2, 87.7%)	72.8 (69.5, 75.8%)	8.5 (6.5, 11.0%)	99.1 (98.4, 99.5%)
	0.0503	78.6 (67.7, 86.5%)	73.2 (70.0, 76.3%)	8.5 (6.5, 11.0%)	99.1 (98.4, 99.5%)
	0.0506	77.1 (65.7, 85.6%)	73.4 (70.1, 76.4%)	8.4 (6.4, 10.9%)	99.0 (98.3, 99.5%)
	0.0509	75.7 (64.5, 84.3%)	73.6 (70.3, 76.6%)	8.3 (6.3, 10.8%)	99.0 (98.2, 99.4%)
	0.0512	74.3 (63.1, 83.0%)	73.9 (70.6, 76.9%)	8.2 (6.3, 10.8%)	98.9 (98.2, 99.4%)
	0.0553	72.9 (61.6, 81.8%)	77.3 (74.3, 79.9%)	9.2 (7.0, 11.9%)	98.9 (98.2, 99.3%)
	0.0577	71.4 (60.1, 80.6%)	79.1 (76.2, 81.7%)	9.7 (7.5, 12.5%)	98.9 (98.1, 99.3%)
	0.0654	70.0 (58.1, 79.7%)	83.4 (80.9, 85.6%)	11.8 (9.1, 15.1%)	98.9 (98.1, 99.3%)
	0.0663	68.6 (57.0, 78.2%)	83.7 (81.3, 85.9%)	11.7 (9.0, 15.1%)	98.8 (98.1, 99.3%)
	0.0767	67.1 (55.9, 76.7%)	87.6 (85.6, 89.5%)	14.6 (11.4, 18.6%)	98.8 (98.1, 99.3%)
	0.0786	65.7 (54.5, 75.4%)	88.1 (86.1, 89.9%)	14.8 (11.6, 18.8%)	98.8 (98.1, 99.2%)
	0.0797	64.3 (53.2, 74.1%)	88.5 (86.5, 90.2%)	15.0 (11.7, 19.1%)	98.7 (98.0, 99.2%)
	0.0835	62.9 (51.8, 72.7%)	89.3 (87.3, 90.9%)	15.6 (12.1, 19.9%)	98.7 (98.0, 99.2%)
	0.0851	61.4 (50.5, 71.4%)	89.7 (87.8, 91.3%)	15.8 (12.2, 20.3%)	98.7 (97.9, 99.1%)
	0.0902	60.0 (49.1, 70.0%)	90.4 (88.6, 91.9%)	16.5 (12.6, 21.3%)	98.6 (97.9, 99.1%)
	0.0910	58.6 (47.2, 69.1%)	90.6 (88.8, 92.1%)	16.4 (12.6, 21.1%)	98.6 (97.8, 99.1%)
	0.0911	57.1 (45.9, 67.7%)	90.6 (88.8, 92.1%)	16.1 (12.3, 20.8%)	98.5 (97.7, 99.0%)
	0.0913	55.7 (44.6, 66.3%)	90.6 (88.8, 92.1%)	15.7 (12.0, 20.4%)	98.5 (97.7, 99.0%)

© 2021 Lind ML et al. *JAMA Network Open.*

	Cut Points	Sensitivity	Specificity	Positive Predictive Value	Negative Predictive Value
	0.0921	54.3 (42.9, 65.3%)	90.8 (89.0, 92.3%)	15.7 (11.9, 20.4%)	98.4 (97.6, 99.0%)
	0.0969	52.9 (41.6, 63.9%)	91.4 (89.8, 92.8%)	16.3 (12.3, 21.2%)	98.4 (97.6, 98.9%)
	0.0984	51.4 (39.9, 62.8%)	91.8 (90.2, 93.2%)	16.5 (12.4, 21.6%)	98.4 (97.5, 98.9%)
	0.0993	50.0 (38.7, 61.3%)	92.1 (90.6, 93.5%)	16.7 (12.6, 22.0%)	98.3 (97.5, 98.9%)
	0.1041	48.6 (37.1, 60.2%)	93.1 (91.6, 94.3%)	18.1 (13.5, 23.8%)	98.3 (97.4, 98.9%)
	0.1045	47.1 (35.8, 58.8%)	93.1 (91.6, 94.3%)	17.7 (13.2, 23.4%)	98.2 (97.4, 98.8%)
	0.1060	45.7 (34.0, 57.9%)	93.3 (91.8, 94.5%)	17.7 (13.0, 23.5%)	98.2 (97.3, 98.8%)
	0.1085	44.3 (33.1, 56.1%)	93.4 (92.0, 94.6%)	17.5 (12.9, 23.3%)	98.2 (97.3, 98.8%)
	0.1146	42.9 (31.8, 54.6%)	94.2 (92.9, 95.2%)	18.9 (13.9, 25.2%)	98.1 (97.2, 98.7%)
	0.1163	41.4 (30.6, 53.1%)	94.4 (93.2, 95.5%)	19.1 (14.0, 25.5%)	98.1 (97.2, 98.7%)
	0.1289	40.0 (29.4, 51.6%)	95.3 (94.2, 96.2%)	21.2 (15.5, 28.3%)	98.1 (97.2, 98.7%)
	0.1293	38.6 (27.9, 50.4%)	95.3 (94.2, 96.2%)	20.6 (14.9, 27.8%)	98.0 (97.1, 98.6%)
	0.1348	37.1 (26.7, 48.9%)	95.8 (94.7, 96.6%)	21.7 (15.6, 29.3%)	98.0 (97.1, 98.6%)
	0.1374	35.7 (25.5, 47.4%)	95.9 (94.9, 96.8%)	21.7 (15.5, 29.6%)	97.9 (97.0, 98.6%)
	0.1418	34.3 (24.3, 45.9%)	96.2 (95.2, 97.0%)	22.0 (15.5, 30.2%)	97.9 (97.0, 98.5%)
	0.1624	32.9 (23.1, 44.3%)	97.1 (96.2, 97.8%)	26.1 (18.5, 35.5%)	97.9 (97.0, 98.5%)
	0.1685	31.4 (21.4, 43.6%)	97.2 (96.3, 97.9%)	26.2 (18.4, 35.8%)	97.8 (96.9, 98.5%)
	0.1702	30.0 (20.2, 42.0%)	97.3 (96.4, 98.0%)	25.9 (17.9, 35.9%)	97.8 (96.9, 98.4%)
	0.1915	28.6 (18.8, 40.9%)	97.7 (97.0, 98.3%)	28.6 (19.5, 39.7%)	97.7 (96.8, 98.4%)
	0.2071	27.1 (17.6, 39.3%)	98.1 (97.4, 98.7%)	31.7 (21.5, 43.9%)	97.7 (96.8, 98.4%)
	0.2163	25.7 (16.5, 37.7%)	98.2 (97.6, 98.7%)	31.6 (21.2, 44.3%)	97.7 (96.7, 98.3%)

© 2021 Lind ML et al. *JAMA Network Open.*

	Cut Points	Sensitivity	Specificity	Positive Predictive Value	Negative Predictive Value
	0.2200	24.3 (15.4, 36.1%)	98.5 (97.9, 99.0%)	34.0 (22.7, 47.5%)	97.6 (96.7, 98.3%)
	0.2243	22.9 (14.3, 34.5%)	98.6 (98.0, 99.0%)	33.3 (22.1, 46.9%)	97.6 (96.6, 98.3%)
	0.2262	21.4 (13.2, 32.9%)	98.6 (98.0, 99.0%)	32.6 (21.1, 46.8%)	97.5 (96.6, 98.2%)
	0.2410	20.0 (12.1, 31.3%)	98.8 (98.2, 99.2%)	34.1 (21.8, 49.1%)	97.5 (96.6, 98.2%)
	0.2524	18.6 (11.0, 29.6%)	99.1 (98.6, 99.5%)	40.6 (25.4, 57.9%)	97.5 (96.5, 98.2%)
	0.2547	17.1 (9.8, 28.1%)	99.2 (98.7, 99.5%)	40.0 (24.5, 57.9%)	97.4 (96.5, 98.1%)
	0.2577	15.7 (8.8, 26.5%)	99.3 (98.8, 99.6%)	40.7 (23.8, 60.2%)	97.4 (96.4, 98.1%)
	0.2621	14.3 (7.8, 24.8%)	99.3 (98.8, 99.6%)	38.5 (21.8, 58.4%)	97.3 (96.4, 98.1%)
	0.2819	12.9 (6.8, 23.0%)	99.5 (99.0, 99.7%)	42.9 (23.5, 64.7%)	97.3 (96.3, 98.0%)
	0.2845	11.4 (5.8, 21.3%)	99.5 (99.0, 99.7%)	40.0 (20.9, 62.7%)	97.3 (96.3, 98.0%)
	0.2970	10.0 (4.8, 19.5%)	99.5 (99.2, 99.8%)	41.2 (21.0, 64.8%)	97.2 (96.3, 97.9%)
	0.2987	8.6 (3.9, 17.8%)	99.5 (99.2, 99.8%)	37.5 (17.9, 62.3%)	97.2 (96.2, 97.9%)
	0.3084	7.1 (3.0, 16.1%)	99.6 (99.3, 99.8%)	38.5 (17.0, 65.6%)	97.1 (96.2, 97.9%)
	0.3237	5.7 (2.2, 14.3%)	99.8 (99.5, 99.9%)	44.4 (17.7, 74.9%)	97.1 (96.1, 97.8%)
	0.3611	4.3 (1.4, 12.4%)	99.9 (99.6, 100%)	50.0 (16.8, 83.2%)	97.1 (96.1, 97.8%)
	0.3807	2.9 (0.7, 10.8%)	99.9 (99.6, 100%)	50.0 (12.3, 87.7%)	97.0 (96.0, 97.8%)
	0.3894	1.4 (0.2, 9.5%)	99.9 (99.6, 100%)	33.3 (4.3, 84.6%)	97.0 (96.0, 97.7%)
	0.5752	0.0 (0.0, 0.0%)	100 (99.7, 100%)	0.0 (0.0, 0.0%)	96.9 (95.9, 97.7%)
SIRS					
	0	100 (100, 100%)	0.0 (0.0, 0.0%)	3.1 (2.4, 3.9%)[c]	-
	1	75.7 (63.5, 84.8%)	34.0 (31.3, 36.8%)	3.5 (2.6, 4.7%)	97.8 (96.1, 98.8%)

© 2021 Lind ML et al. *JAMA Network Open.*

	Cut Points	Sensitivity	Specificity	Positive Predictive Value	Negative Predictive Value
	2	54.3 (41.9, 66.2%)	75.9 (73.6, 78.0%)	6.6 (4.8, 9.1%)	98.1 (97.1, 98.8%)
	3	10.0 (5.0, 19.2%)	95.5 (94.5, 96.3%)	6.5 (3.1, 13.2%)	97.1 (96.1, 97.8%)
	4	1.4 (0.2, 9.3%)	99.8 (99.5, 99.9%)	16.7 (2.3, 63.1%)	97.0 (96.0, 97.7%)
qSOFA					
	0	100 (100, 100%)	0.0 (0.0, 0.0%)	3.1 (2.4, 3.9%)[c]	-
	1	18.6 (11.2, 29.2%)	81.5 (79.6, 83.3%)	3.1 (1.8, 5.2%)	96.9 (95.8, 97.8%)
	2	7.1 (3.0, 16.1%)	99.5 (99.0, 99.7%)	29.4 (13.2, 53.3%)	97.1 (96.2, 97.9%)
	3	1.4 (0.2, 9.5%)	99.9 (99.6, 100%)	33.3 (4.3, 84.6%)	97 (96.0, 97.7%)
NEWS					
	0	100 (100, 100%)	0.0 (0.0, 0.0%)	3.1 (2.4, 3.9%)[c]	
	1	87.1 (77.2, 93.1%)	18.2 (16.3, 20.2%)	3.3 (2.5, 4.3%)	97.8 (95.6, 98.9%)
	2	68.6 (57.3, 78.0%)	41.6 (39.2, 44.1%)	3.6 (2.7, 4.8%)	97.7 (96.3, 98.5%)
	3	48.6 (37.7, 59.6%)	64.2 (61.8, 66.5%)	4.1 (2.9, 5.7%)	97.5 (96.4, 98.3%)
	4	31.4 (21.5, 43.4%)	81.9 (80.1, 83.6%)	5.2 (3.4, 7.8%)	97.4 (96.4, 98.2%)
	5	21.4 (13.4, 32.4%)	92.2 (90.9, 93.4%)	8.0 (4.9, 12.8%)	97.4 (96.4, 98.1%)
	6	17.1 (10.0, 27.7%)	97.2 (96.4, 97.9%)	16.4 (9.7, 26.5%)	97.4 (96.4, 98.1%)
	7	8.6 (3.9, 17.9%)	98.7 (98.0, 99.1%)	17.1 (7.8, 33.7%)	97.2 (96.2, 97.9%)
	8	5.7 (2.2, 14.3%)	99.2 (98.7, 99.5%)	19.0 (7.3, 41.4%)	97.1 (96.1, 97.8%)
	9	4.3 (1.4, 12.6%)	99.6 (99.3, 99.8%)	27.3 (9.0, 58.6%)	97.1 (96.1, 97.8%)
	10	2.9 (0.7, 10.8%)	99.9 (99.6, 100%)	40.0 (10.0, 80.0%)	97.0 (96.0, 97.8%)
	11				
	12				
	13	0.0 (0.0, 5.1%)	99.9 (99.7, 100%)	0.0 (0.0, 0.0%)	96.9 (95.9, 97.7%)

© 2021 Lind ML et al. *JAMA Network Open.*

	Cut Points	Sensitivity	Specificity	Positive Predictive Value	Negative Predictive Value
	14	0.0 (0.0, 5.1%)	99.9 (99.7, 100%)	0.0 (0.0, 0.0%)	96.9 (95.9, 97.7%)
	15[b]	-	-	-	-
	16[b]	-	-	-	-
	17[b]	-	-	-	-
	18[b]	-	-	-	-
	19[b]	-	-	-	-
	20[b]	-	-	-	-
[a] 95% Confidence Intervals estimated using Clopper Pearson methods					
[b] No PBIs accompanied within these scores in the validation dataset					

There were multiple NEWS scores that did not appear in our validation dataset, namely higher ones (15-2). This is likely the fact that we are observed a large amount of missingness in predictors included in this tool and we imputed missingness using a single normal value. For example, we observed >90% missingness within GCS and >60% missingness within oxygen saturation (Spo2).

© 2021 Lind ML et al. *JAMA Network Open.*

eTable 5. Categorical Reclassification Index Comparing Risk Classification of Full Predictor Tool (SHBSL) With Other Examined Tools

	NRI	95% CI	Pvalue
Clinical Factor Specific - C-SHBSL[a]	0.37	0.20 - 0.54	<0.001
SIRS[b]	0.52	0.34 - 0.69	<0.001
qSOFA[b]	0.31	0.13 - 0.49	<0.001
NEWS[b]	0.35	0.20 - 0.49	<0.001
[a]Categories defined as quartiles of C-SBHSL risk prediction			
[b]Categories defined as risk probabilities categories estimated using single variable logistic regression			

© 2021 Lind ML et al. *JAMA Network Open.*

eFigure 5. Summary of Prediction Scores by Patient Location

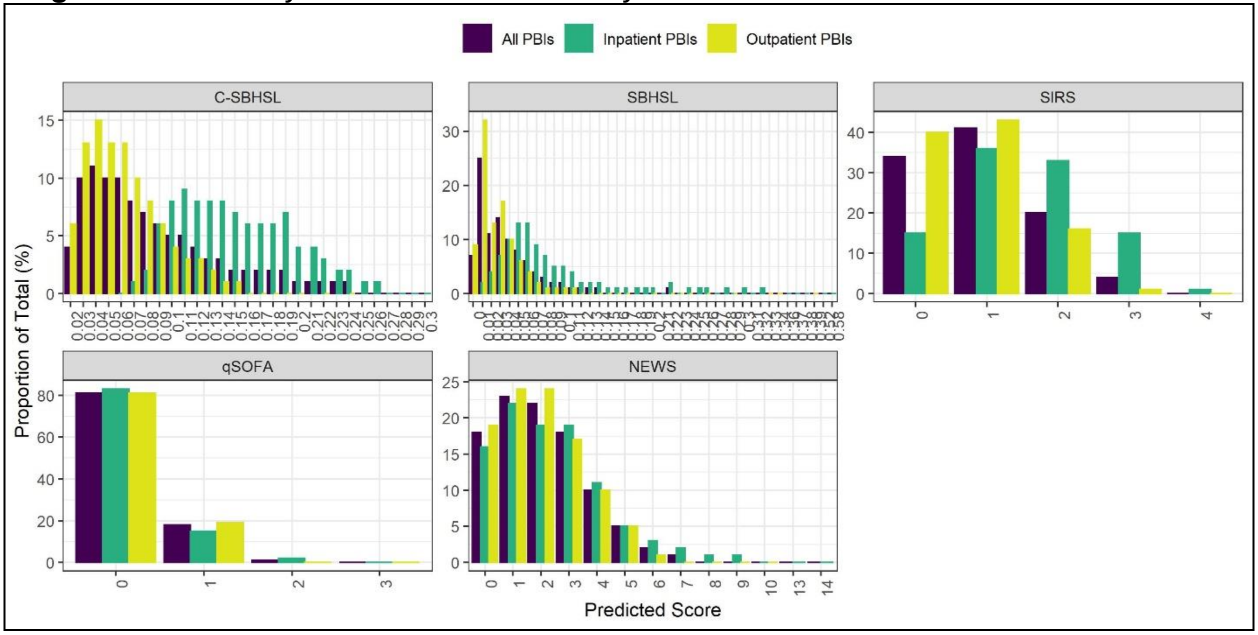

© 2021 Lind ML et al. *JAMA Network Open.*

eAppendix 5. Ensemble Selection Rational

The cross validated area under the curve (cvAUC) estimates for each user supplied algorithm and the resulting Super Learner for the full predictor (SHBSL) and clinical factor-specific predictor (C-SHBSL) prediction tools. The name structure for is algorithm is as follows: SL refers to the fact that the algorithms are running within the super learner framework, algorithm name (detailed list above), screen (screen.corP_strick or screen.glmnet) or absence of screen (All).

Despite the Super Learner ensembles having slightly lower cvAUCs than (screened - SHBSL and base – C-SHBSL) random forest with balanced groups of 100 samples each, we chose to present the ensembles as our final models. We made this decision because the ensembles performed essentially as well as the random forest (<1 AUC percentage point difference) and they provide the least biased option for future updates. Because excessive testing has shown that, in small data settings, the Super Learner performs essentially as well as any provided algorithm[4] (as we saw in our data), we feel comfortable assuming that if, upon future parameter updating, the best fit provided algorithm is no longer the group-matched random forest, the Super Learner will still perform essentially as well as (if not better than) the best provided algorithm.

© 2021 Lind ML et al. *JAMA Network Open.*

eFigure 6. Cross-Validated Area Under the Curve Estimates of Super Learner and User-Supplied Algorithms

A. SHBSL – Full Predictor Tool

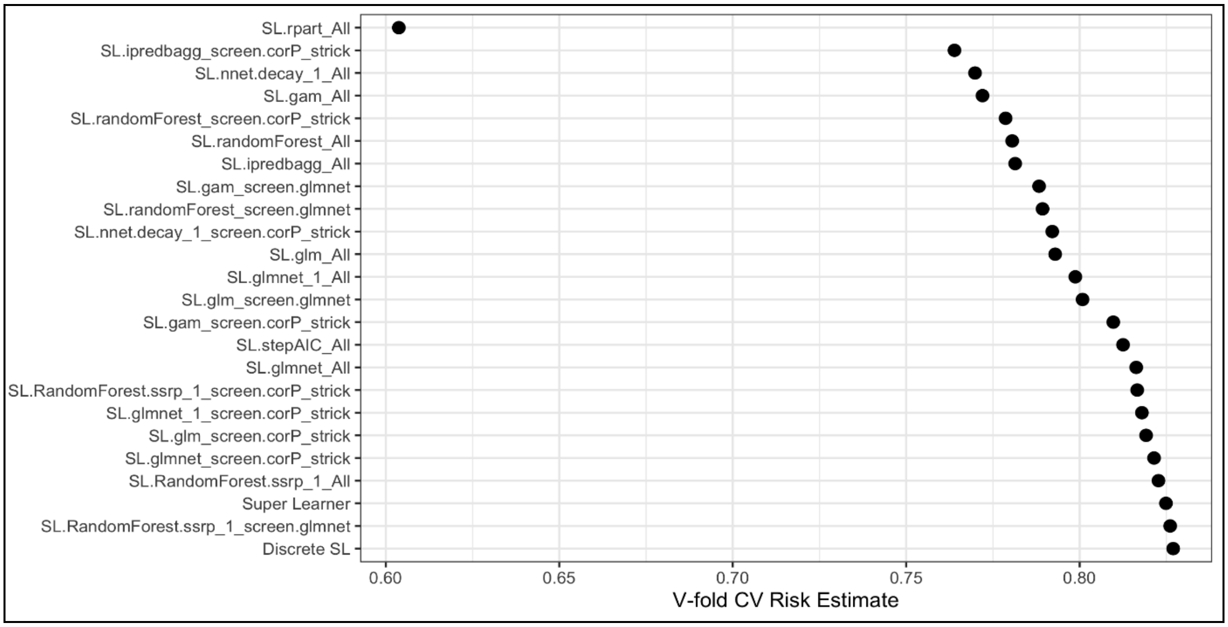

B. C-SHBSL – Clinical Factor-Specific Tool

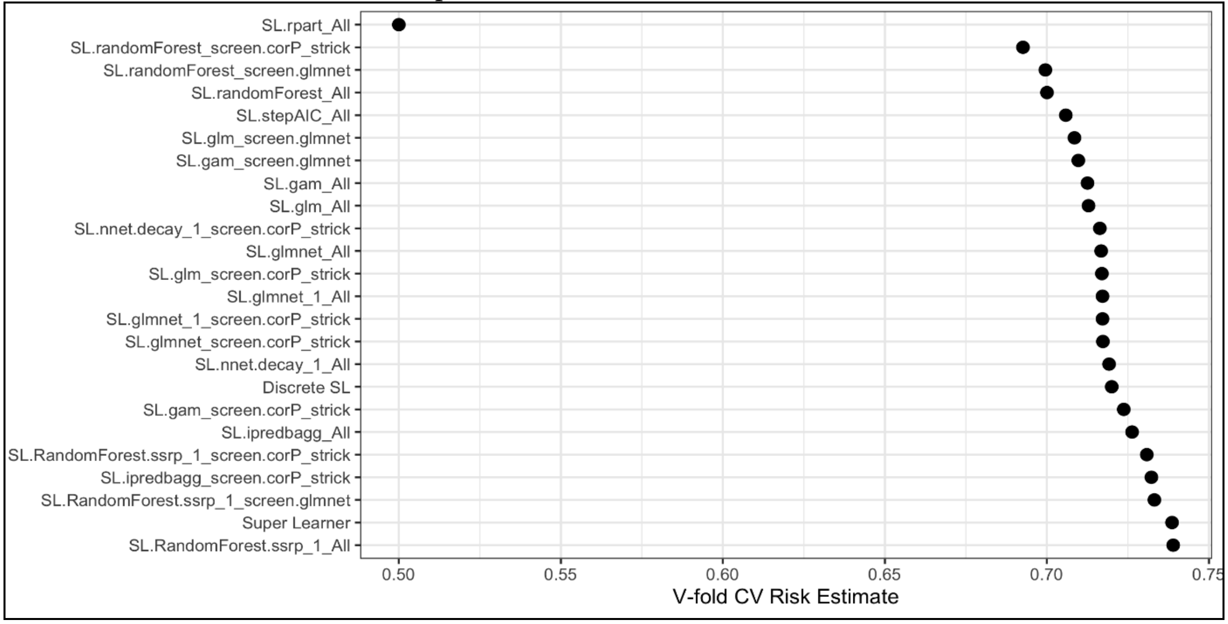

© 2021 Lind ML et al. *JAMA Network Open.*

eTable 6. Cross-Validated and Bootstrapped Area Under the Curve Estimates for High–Sepsis Risk Bacteremia[a]

	AUC	95% CIs
Clinical Factor-Specific - C-SHBSL [a]	0.74	0.59 - 0.86
Full Predictor - SHBSL[a]	0.85	0.70 - 0.91
SIRS (2+)[b]	0.64	0.52 - 0.77
qSOFA (2+)[b]	0.51	0.44 - 0.60
NEWS[b]	0.60	0.50 - 0.72
[a] cvAUC estimates were generated using nested 10-fold cross validation		
[b] 1000 Bootstrapped Estimates		

© 2021 Lind ML et al. *JAMA Network Open.*

eTable 7. Calibration and Observed vs Estimated High–Sepsis Risk Bacteremia Probabilities

Observed bacterial sepsis (%)	3.1%
Sepsis prediction (mean (IQR))	
Clinical Factor Specific - C-SHBSL [a]	0.031 (0.013, 0.033)
Full Predictor-SHBSL[a]	0.031 (0.013, 0.024)
SIRS	0.031 (0.016, 0.048)
qSOFA	0.031 (0.028, 0.028)
NEWS	0.031 (0.022, 0.035)
[a] Performed in the validation dataset	

© 2021 Lind ML et al. *JAMA Network Open.*

eAppendix 6. Sensitivity Analyses

Our tools were developed using data from a single center and may be biased towards data collection practices of that center. In the absence of external data (another center's) data for validation at the time of manuscript development, we performed numerous sensitivity analyses to better understand the generalizability of our developed tools. Each sensitivity analysis was conducted among patients randomized to the held-out validation dataset. We estimated each tools AUROC and sensitivity and specificity and the following cut-points: 2+ for SIRS and qSOFA, 7+ for NEWS, and Upper-Left method selected optimal cut-point for our tools (selected in the primary analysis held-out validation data – SHBSL: 5.0%, C-SHBSL: 9.3%).

Missing Data

Because vitals and laboratory measure missingness may differ between clinical staff, our tool may be biased towards the missingness patterns of our center. We performed numerous sensitivity analyses to examine the robustness of our tool's superiority over existing tools under varying missingness scenarios. Along with testing the robustness of our results to missing data patterns, these analyses also provide insight into the predictor collection requirements of each tool (ie. does each tool require complete data to run optimally or can it produce similar estimations under varying amounts of missingness). Specifically, we examined the predictive value of each tool under the following scenarios:

1. Complete Case (restricted to only samples with full data present)
2. Partial Missingness (restricted to samples with at least one missing sample)
3. Average Missingness (restricted to samples with at least three (the average observed number of missing measurements) missing measurements
4. Single Value from Observed Range Imputation (values within the observed predictor range were selected at random for each observation)

© 2021 Lind ML et al. *JAMA Network Open.*

eTable 8. Predictive Ability of Examined Prognostic Tools for High–Sepsis Risk Bacteremia Among Allogeneic HCT Recipients with PBIs Under Varying Missing Data Assumptions

	AUROC[b,c]	PR-AUC[b]	Sensitivity[d]	Specificity[d]
Complete Case				
Clinical Factor Specific - C-SHBSL[a]	0.76 (0.67, 0.85)	0.08	75.0 (51.7, 89.4%)	59.5 (55.3, 63.6%)
Full Predictor-SHBSL[a]	0.81 (0.71, 0.90)	0.11	70.0 (48.5, 85.3%)	72.3 (68.2, 76.1%)
SIRS (2+)	0.60 (0.46, 0.75)	0.07	55.0 (32.5, 75.7%)	68.8 (65.2, 72.2%)
qSOFA (2+)	0.59 (0.49, 0.69)	0.09	10.0 (2.5, 32.6%)	99.0 (98.1, 99.5%)
NEWS (4+)	0.57 (0.40, 0.74)	0.06	10.0 (2.5, 32.6%)	98.4 (97.5, 99.0%)
Partial Missing for All Potential Infections				
Clinical Factor Specific - C-SHBSL[a]	0.73 (0.64, 0.81)	0.23	66.0 (48.5, 80.0%)	71.0 (67.6, 74.2%)
Full Predictor-SHBSL[a]	0.86 (0.81, 0.91)	0.25	80.0 (67.9, 88.3%)	74.5 (70.6, 78.2%)
SIRS (2+)	0.67 (0.59, 0.76)	0.08	54.0 (40.3, 67.1%)	81.9 (79.5, 84.1%)
qSOFA (2+)	0.45 (0.39, 0.51)	0.11	6.0 (1.9, 17.2%)	99.8 (99.3, 100%)
NEWS (4+)	0.57 (0.48, 0.65)	0.10	8.0 (3.0, 19.7%)	99.1 (98.3, 99.5%)
Average Missing for All Potential Infections				
Clinical Factor Specific - C-SHBSL[a]	0.64 (0.51, 0.77)	0.17	50.0 (29.3, 70.7%)	68.6 (64.7, 72.2%)
Full Predictor-SHBSL[a]	0.85 (0.78, 0.92)	0.21	76.9 (57.3, 89.2%)	72.9 (68.4, 77.0%)
SIRS (2+)	0.64 (0.52, 0.75)	0.05	42.3 (26.6, 59.8%)	85.4 (82.8, 87.7%)
qSOFA (2+)	0.43 (0.35, 0.51)	0.12	3.8 (0.5, 23.0%)	100 (99.6, 100%)[e]
NEWS (4+)	0.61 (0.50, 0.72)	0.04	3.8 (0.5, 23.0%)	99.3 (98.5, 99.7%)
Single Value from Observed Range Imputation				
Clinical Factor Specific - C-SHBSL[a]	0.67 (0.60, 0.74)	0.09	68.6 (55.6, 79.1%)	55.3 (52.5, 58.1%)

© 2021 Lind ML et al. *JAMA Network Open.*

Full Predictor-SHBSL[a]	0.84 (0.79, 0.88)	0.15	80.0 (67.9, 88.3%)	73.7 (70.7, 76.5%)
SIRS (2+)	0.60 (0.54, 0.67)	0.05	65.7 (53.7, 76.0%)	52.6 (49.9, 55.4%)
qSOFA (2+)	0.48 (0.41, 0.54)	0.07	27.1 (17.7, 39.2%)	72.9 (70.3, 75.3%)
NEWS (4+)	0.51 (0.44, 0.57)	0.03	45.7 (33.5, 58.4%)	59.7 (56.8, 62.5%)

[a] Based on Upper-Left Selected Cut-points from primary validation analysis (C-SHBSL: 9.3%; SHBSL: 5.0%)

[b] AUROC: area under the receiver operating characteristic curve, PR-AUC: area under the precision recall curve

[c] 95% Confidence Interval estimated using 2000 stratified bootstrapped replicates

[d] 95% Confidence Intervals estimated using generalized estimating equations with robust standard errors

[e] 95% Confidence Intervals estimated using Clopper Pearson methods (perfect prediction [aka. 100%] prevented uncertainty estimation from generalized estimating equation)

© 2021 Lind ML et al. *JAMA Network Open.*

Race Stratified

Despite the Fred Hutchinson Cancer Research Center / Seattle Cancer Care Alliance (FHCRC/SCCA) having a large catchment area in the Northwest region of the United States, its transplant patient population is largely non-Hispanic white and may not reflect (racially/ethnically) the national transplant population. This is concerning within the context of sepsis prediction modeling because of evidence suggesting that there are racial/ethnic differences in both the rate of transplant complications and sepsis.[5–8] To examine the generalizability of our tool to a more racially/ethnically diverse population, we estimated the predictive value of our tool stratified by race/ethnicity. Due to limited data for non-white racial/ethnic groups, we examined the following stratification:

1. non-Hispanic White
2. Other

eTable 9. Predicative Ability of Sepsis Prognosis Tools Among Varying Racial/Ethnic Groups

	**AUROC[b,c]**	**PR-AUC[b]**	**Sensitivity[d]**	**Specificity[d]**
non-Hispanic White				
Clinical Factor Specific - C-SHBSL[a]	0.72 (0.64, 0.80)	0.17	63.0 (45.3, 77.8%)	66.8 (63.4, 69.9%)
Full Predictor-SHBSL[a]	0.86 (0.82, 0.91)	0.15	82.6 (71.3, 90.1%)	74.2 (70.6, 77.5%)
SIRS (2+)	0.64 (0.55, 0.72)	0.07	52.2 (37.8, 66.2%)	76.2 (73.5, 78.7%)
qSOFA (2+)	0.50 (0.44, 0.56)	0.07	6.5 (2.1, 18.8%)	99.5 (98.9, 99.8%)
NEWS (4+)	0.60 (0.51, 0.69)	0.08	10.9 (4.5, 23.9%)	99.2 (98.6, 99.5%)
Other				
Clinical Factor Specific - C-SHBSL[a]	0.72 (0.61, 0.83)	0.10	66.7 (45.3, 82.9%)	68.0 (61.6, 73.8%)
Full Predictor-SHBSL[a]	0.81 (0.71, 0.91)	0.36	70.8 (48.5, 86.2%)	72.8 (66.1, 78.6%)
SIRS (2+)	0.63 (0.51, 0.76)	0.06	58.3 (35.5, 78.1%)	75.0 (71.0, 78.7%)
qSOFA (2+)	0.51 (0.42, 0.60)	0.07	8.3 (2.2, 27.1%)	99.4 (98.3, 99.8%)
NEWS (4+)	0.56 (0.44, 0.69)	0.07	8.3 (2.2, 27.1%)	97.8 (96.2, 98.7%)

[a] Based on Upper-Left Selected Cut-points from primary validation analysis (C-SHBSL: 9.3%; SHBSL: 5.0%)

[b] AUROC: area under the receiver operating characteristic curve, PR-AUC: area under the precision recall curve

[c] 95% Confidence Interval estimated using 2000 stratified bootstrapped replicates

[d] 95% Confidence Intervals estimated using generalized estimating equations with robust standard errors

© 2021 Lind ML et al. *JAMA Network Open.*

Window of Measurement Collection

We developed tools to provide decision support at the time of culture collection and based on our predictor measurement inclusion defined the run time of the tools as 2 hours following culture collection. This time period was selected because predictor measurements were commonly collected within the first few hours following culture collection and we considered these measures to be associated with the collection of the culture. However, in practice, it may be desirable to run these tools closer in time to the culture. For this reason, we estimated the AUC, sensitivity, and specificities of each examined tools using data from the 24 hours and:

1. 1 hours prior
2. 0.5 hours prior
3. 0 hours prior

eTable 10. Predicative Ability of Sepsis Prognosis Tools Under Varying Factor Measurement Collection Time Windows

	AUROC[b,c]	PR-AUC[b]	Sensitivity[d]	Specificity[d]
0 Hours				
Clinical Factor Specific - C-SHBSL[a]	0.7 (0.63, 0.76)	0.11	61.4 (48.5, 72.9%)	67.6 (64.7, 70.4%)
Full Predictor-SHBSL[a]	0.85 (0.81, 0.9)	0.20	81.4 (71.4, 88.5%)	72.4 (69.3, 75.4%)
SIRS (2+)	0.63 (0.56, 0.7)	0.07	41.4 (29.8, 54.1%)	81.5 (79.4, 83.4%)
qSOFA (2+)	0.56 (0.5, 0.61)	0.10	5.7 (2.2, 14.3%)	99.7 (99.3, 99.9%)
NEWS (4+)	0.63 (0.56, 0.7)	0.07	5.7 (2.2, 14.3%)	98.9 (98.3, 99.3%)
0.5 Hours				
Clinical Factor Specific - C-SHBSL[a]	0.78 (0.75, 0.82)	0.18	73.9 (67.1, 79.8%)	67.0 (65.3, 68.6%)
Full Predictor-SHBSL[a]	0.93 (0.92, 0.95)	0.39	93.3 (88.9, 96%)	75.3 (73.6, 77.0%)
SIRS (2+)	0.61 (0.58, 0.65)	0.05	42.4 (36.0, 49.2%)	78.6 (77.4, 79.7%)
qSOFA (2+)	0.50 (0.48, 0.53)	0.05	2.5 (1.1, 5.5%)	99.5 (99.4, 99.7%)
NEWS (4+)	0.60 (0.57, 0.64)	0.05	3.8 (2.0, 7.1%)	98.9 (98.5, 99.1%)
1 Hour				
Clinical Factor Specific - C-SHBSL[a]	0.74 (0.68, 0.80)	0.13	65.7 (51.2, 77.8%)	67.0 (64.0, 69.8%)
Full Predictor-SHBSL[a]	0.85 (0.81, 0.90)	0.20	78.6 (65.9, 87.4%)	73.4 (70.2, 76.4%)
SIRS (2+)	0.62 (0.55, 0.69)	0.06	45.7 (33.5, 58.4%)	77.3 (75.1, 79.4%)

© 2021 Lind ML et al. *JAMA Network Open.*

qSOFA (2+)	0.54 (0.49, 0.60)	0.09	7.1 (3.0, 16.1%)	99.5 (99.0, 99.7%)
NEWS (4+)	0.61 (0.54, 0.69)	0.07	7.1 (3.0, 16.1%)	98.6 (98.0, 99.1%)

[a] Based on Upper-Left Selected Cut-points from primary validation analysis (C-SHBSL: 9.3%; SHBSL: 5.0%)

[b] AUROC: area under the receiver operating characteristic curve, PR-AUC: area under the precision recall curve

[c] 95% Confidence Interval estimated using 2000 stratified bootstrapped replicates

[d] 95% Confidence Intervals estimated using generalized estimating equations with robust standard errors

© 2021 Lind ML et al. *JAMA Network Open.*

Culture Collection Practices

Our tools are designed to supply decision support at the time of blood culture collection and are likely biased towards the culture collection practices at our center. While we cannot retrospectively change the culture collection practices of our center to examine the generalizability of our tools, we were able to adjust the cultures we included and include a proportion of non-culture time to simulate a "what if cultures had been collected" scenario.

1. Excluding Surveillance Cultures - Surveillance status was not perfectly recorded, and this sensitivity analysis was performed among cultures with recorded reasons other than "surveillance." While this reduces the number of surveillance cultures included in our analysis, it may not fully remove them.
2. Percent non-Culture 24 Hours: To the observed PBIs in the validation data (2286), we added additional periods of 24-hour follow-up. The 24-hour (one full calendar day) data collection periods were randomly selected among calendar days between transplant and end of post-transplant follow-up. 24-hour periods within 3 days of a culture collection and during follow-up cultures periods were excluded.
    a. Added an additional 114 (or 5%) non-Culture 24 hours
    b. Added an additional 229 (or 10%) non-Culture 24 hours

eTable 11. Predictive Ability of Sepsis Prognosis Tools Under Varying Culture Collection and Potential Infection Restriction Definitions

	**AUROC[b,c]**	**PR-AUC[b]**	**Sensitivity[d]**	**Specificity[d]**
Cultures Excluding Survellience[b]				
Clinical Factor Specific - C-SHBSL[a]	0.71 (0.64, 0.78)	0.14	66.2 (52.8, 77.3%)	61.7 (57.9, 65.3%)
Full Predictor-SHBSL[a]	0.83 (0.79, 0.88)	0.21	78.5 (67.0, 86.7%)	74.0 (70.9, 76.8%)
SIRS (2+)	0.64 (0.57, 0.70)	0.08	56.9 (43.9, 69.0%)	71.0 (68.2, 73.7%)
qSOFA (2+)	0.56 (0.51, 0.62)	0.10	9.2 (4.1, 19.3%)	99.1 (98.4, 99.5%)
NEWS (4+)	0.62 (0.55, 0.70)	0.09	10.8 (5.1, 21.2%)	98.1 (97.0, 98.7%)
5% non-Culture 24 hours				
Clinical Factor Specific - C-SHBSL[a]	0.73 (0.67, 0.8)	0.14	68.6 (54.0, 80.2%)	66.9 (64.1, 69.6%)
Full Predictor-SHBSL[a]	0.85 (0.81, 0.9)	0.21	81.4 (69.5, 89.4%)	73.4 (70.2, 76.2%)
SIRS (2+)	0.63 (0.57, 0.7)	0.06	48.6 (36.2, 61.1%)	76.2 (74.0, 78.3%)
qSOFA (2+)	0.55 (0.5, 0.61)	0.09	7.1 (3.0, 16.1%)	99.4 (99.0, 99.7%)
NEWS (4+)	0.63 (0.56, 0.7)	0.08	10.0 (4.8, 19.5%)	98.9 (98.3, 99.3%)
10% non-Culture 24 hours				

Clinical Factor Specific - C-SHBSL[a]	0.86 (0.83, 0.89)	0.38	83.2 (76.9, 88.1%)	66.6 (64.9, 68.1%)
Full Predictor-SHBSL[a]	0.94 (0.93, 0.96)	0.45	94.1 (89.8, 96.7%)	75.8 (74.1, 77.3%)
SIRS (2+)	0.63 (0.60, 0.67)	0.06	49.6 (42.8, 56.3%)	76.6 (75.4, 77.8%)
qSOFA (2+)	0.52 (0.50, 0.55)	0.05	3.4 (1.7, 6.6%)	99.5 (99.3, 99.6%)
NEWS (4+)	0.6 (0.57, 0.64)	0.05	5.9 (3.5, 9.7%)	98.7 (98.3, 99.1%)

[a] Based on Upper-Left Selected Cut-points from primary validation analysis (C-SHBSL: 9.3%; SHBSL: 5.0%)

[b] AUROC: area under the receiver operating characteristic curve, PR-AUC: area under the precision recall curve

[c] 95% Confidence Interval estimated using 2000 stratified bootstrapped replicates

[d] 95% Confidence Intervals estimated using generalized estimating equations with robust standard errors

© 2021 Lind ML et al. *JAMA Network Open.*

Antibiotic Influence on Culture Results

We carefully selected our definition of bacterial sepsis for our tool development but understand that culture confirmation of a blood borne infection comes with limitations. A large limitation being that recent antibiotic use may impact culture results. We addressed this in tool development and the primary evaluation by excluding cultures collected within 3 days of antibiotic use but excluded our center's primary prophylaxis, levofloxacin. To further account for the potential impact of recent antibiotic use, we performed the following sensitivity analysis:

1. All Cultures Regardless of Recent Antibiotic Use
2. No Antibiotic Use in Last 3 Days (Including Levofloxacin)
3. No Use in Last 7 Days (Excluding Levofloxacin)

eTable 12. Predictive Ability of Sepsis Prognosis Tools Under Varying Recent Antibiotic Definitions

	AUROC[b,c]	PRAUC[b]	Sensitivity[d]	Specificity[d]
All Cultures Regardless of Recent Antibiotic Use				
Clinical Factor Specific - C-SHBSL[a]	0.64 (0.59, 0.68)	0.06	72.3 (62.5, 80.4%)	52.8 (49.4, 56.1%)
Full Predictor-SHBSL[a]	0.81 (0.78, 0.84)	0.15	75.9 (69.1, 81.6%)	69.6 (66.7, 72.4%)
SIRS (2+)	0.56 (0.52, 0.60)	0.04	50.4 (41.9, 58.8%)	61.3 (58.3, 64.2%)
qSOFA (2+)	0.51 (0.47, 0.55)	0.05	7.1 (3.8, 12.9%)	97.2 (95.6, 98.3%)
NEWS (4+)	0.55 (0.50, 0.60)	0.04	9.9 (6.0, 16.1%)	93.7 (91.3, 95.4%)
No Use[b] in Last 3 Days (Including Levofloxacin)				
Clinical Factor Specific - C-SHBSL[a]	0.67 (0.59, 0.75)	0.10	56.6 (41.0, 71.0%)	68.4 (65.5, 71.2%)
Full Predictor-SHBSL[a]	0.83 (0.78, 0.88)	0.13	69.8 (55.7, 81.0%)	76.2 (73.1, 79.1%)
SIRS (2+)	0.60 (0.52, 0.69)	0.07	41.5 (28.1, 56.4%)	78.2 (75.8, 80.5%)
qSOFA (2+)	0.57 (0.50, 0.63)	0.09	7.5 (2.8, 18.6%)	99.6 (99.2, 99.8%)
NEWS (4+)	0.59 (0.50, 0.68)	0.06	7.5 (2.8, 18.6%)	98.6 (97.9, 99.1%)
No Use[b] in Last 7 Days (Excluding Levofloxacin)				
Clinical Factor Specific - C-SHBSL[a]	0.77 (0.70, 0.83)	0.14	70.0 (57.1, 80.3%)	68.1 (65.2, 70.9%)
Full Predictor-SHBSL[a]	0.86 (0.82, 0.91)	0.24	85.0 (73.2, 92.2%)	71.5 (68.1, 74.7%)
SIRS (2+)	0.66 (0.59, 0.73)	0.07	55.0 (41.7, 67.6%)	76.7 (74.3, 78.9%)

© 2021 Lind ML et al. *JAMA Network Open.*

qSOFA (2+)	0.54 (0.49, 0.60)	0.08	5.0 (1.6, 14.3%)	99.6 (99.1, 99.8%)
NEWS (4+)	0.62 (0.54, 0.70)	0.07	6.7 (2.5, 16.4%)	99.2 (98.7, 99.5%)

[a] Based on Upper-Left Selected Cut-points from primary validation analysis (C-SHBSL: 9.3%; SHBSL: 5.0%)

[b] AUROC: area under the receiver operating characteristic curve, PR-AUC: area under the precision recall curve

[c] 95% Confidence Interval estimated using 2000 stratified bootstrapped replicates

[d] 95% Confidence Intervals estimated using generalized estimating equations with robust standard errors

eReferences

1. Hämäläinen S, Kuittinen T, Matinlauri I, Nousiainen T, Koivula I, Jantunen E. Severe sepsis in autologous stem cell transplant recipients: Microbiological aetiology, risk factors and outcome. *Scandinavian Journal of Infectious Diseases*. 2009;41(1):14-20. doi:10.1080/00365540802454706

2. Póvoa P, Almeida E, Moreira P, et al. C-reactive protein as an indicator of sepsis. *Intensive Care Med*. 1998;24(10):1052-1056.

3. Abe R, Oda S, Sadahiro T, et al. Gram-negative bacteremia induces greater magnitude of inflammatory response than Gram-positive bacteremia. *Crit Care*. 2010;14(2):R27. doi:10.1186/cc8898

4. Laan MJ van der, Rose S. *Targeted Learning: Causal Inference for Observational and Experimental Data*. 2011 edition. Springer; 2011.

5. Majhail NS, Nayyar S, Santibañez MEB, Murphy EA, Denzen EM. Racial disparities in hematopoietic cell transplantation in the United States. *Bone Marrow Transplant*. 2012;47(11). doi:10.1038/bmt.2011.214

6. Jones JM, Fingar KR, Miller MA, et al. Racial Disparities in Sepsis-Related In-Hospital Mortality: Using a Broad Case Capture Method and Multivariate Controls for Clinical and Hospital Variables, 2004-2013. *Crit Care Med*. 2017;45(12):e1209-e1217. doi:10.1097/CCM.0000000000002699

7. Moore JX, Donnelly JP, Griffin R, et al. Black-white racial disparities in sepsis: a prospective analysis of the REasons for Geographic And Racial Differences in Stroke (REGARDS) cohort. *Crit Care*. 2015;19(1). doi:10.1186/s13054-015-0992-8

8. DiMeglio M, Dubensky J, Schadt S, Potdar R, Laudanski K. Factors Underlying Racial Disparities in Sepsis Management. *Healthcare (Basel)*. 2018;6(4). doi:10.3390/healthcare6040133

© 2021 Lind ML et al. *JAMA Network Open.*
